# Genetic risk variants for metabolic traits in Arab populations

**DOI:** 10.1038/srep40988

**Published:** 2017-01-20

**Authors:** Prashantha Hebbar, Naser Elkum, Fadi Alkayal, Sumi Elsa John, Thangavel Alphonse Thanaraj, Osama Alsmadi

**Affiliations:** 1 Dasman Diabetes Institute, P.O. Box 1180, Dasman 15462, Kuwait

## Abstract

Despite a high prevalence of metabolic trait related diseases in Arabian Peninsula, there is a lack of convincingly identified genetic determinants for metabolic traits in this population. Arab populations are underrepresented in global genome-wide association studies. We genotyped 1965 unrelated Arab individuals from Kuwait using Cardio-MetaboChip, and tested SNP associations with 13 metabolic traits. Models based on recessive mode of inheritance identified Chr15:40531386-rs12440118/*ZNF106*/W->R as a risk variant associated with glycated-hemoglobin at close to ‘genome-wide significant’ p-value and five other risk variants ‘nominally’ associated (p-value ≤ 5.45E-07) with fasting plasma glucose (rs7144734/[*OTX2-AS1,RPL3P3*]) and triglyceride (rs17501809/*PLGRKT*; rs11143005/*LOC105376072*; rs900543/[*THSD4,NR2E3*]; and Chr12:101494770/*IGF1*). Furthermore, we identified 33 associations (30 SNPs with 12 traits) with ‘suggestive’ evidence of association (p-value < 1.0E-05); 20 of these operate under recessive mode of inheritance. Two of these ‘suggestive’ associations (rs1800775-*CETP*/HDL; and rs9326246-*BUD13*/TGL) showed evidence at genome-wide significance in previous studies on Euro-centric populations. Involvement of many of the identified loci in mediating metabolic traits was supported by literature evidences. The identified loci participate in critical metabolic pathways (such as Ceramide signaling, and Mitogen-Activated Protein Kinase/Extracellular Signal Regulated Kinase signaling). Data from Genotype-Tissue Expression database affirmed that 7 of the identified variants differentially regulate the up/downstream genes that mediate metabolic traits.

Prevalence of obesity and Type 2 diabetes (T2DM) is enormously rising in the Arabian Peninsula^1^. Rapid transitions in diet practices and lifestyle factors have resulted in high incidence of lifestyle disorders in the Middle East, making the region an epicenter of the escalating epidemics of diabetes. Our recent study using nationwide health data estimated the prevalence of T2DM to be 25% among Kuwaiti natives^2^. Till date, not many genome-wide association (GWA) studies have been conducted on populations from the Arabian Peninsula. Few that have been reported include: (i) a T2DM case-control study on Saudi Arabian population that validated only some (*WFS1, JAZF1, SLC30A8, CDKN2A/B, TCF7L2, KCNQ1, HMG20A, HNF4A* and *DUSP9*) of the T2DM genes implicated in European population^3^; however, the study was not adjusted for population stratification. (ii) a T2DM case-control study on Lebanese population that replicated only two (*CDKAL1* and *TCF7L2*) of the genes implicated in European population^4^; and (iii) a study from the United Arab Emirates^5^ on an extended pedigree (consisting of 178 Arab individuals) that implicated association of *KCTD8, COX7B2* and *GABRA4* genes with T2DM for the first time; the implicated markers have not so far been replicated in any other population.

Indigenous Arabs are descendants of the earliest split from ancient Eurasian populations^6^. A significant portion of the ancestry of indigenous Arabs can be traced back to ancient lineages of the Arabian Peninsula^6^,^7^. These observations have implications for disease genetics studies in the region - for complex diseases such as T2DM, the associated genetic variants in Arab populations would not *a priori* be expected to be the same (or to exert similar effect) as those discovered in European populations. For example, although rs7903146 and rs12255372 variants in *TCF7L2* gene show strong associations with T2DM in most populations, they show weak or no association in Arab populations^8^.

Furthermore, there exists a high level of inbreeding in the Arab region due to the practice of consanguineous marriages that are often between first cousins. This has led to excess of homozygosity for autosomal recessiveness^9^,^10^. It is estimated that nearly two-thirds of genetically transmitted diseases in Arab patients follow an autosomal recessive mode of inheritance^11^. Consanguinity can exert an influence on the etiology of complex disorders if rare autosomal recessive alleles (as opposed to alleles that are common in the gene pool) are causally implicated^12^. Therefore, it is possible to hypothesize that inbreeding helps in aggregating risk alleles and thereby in inducing possible cumulative recessive effects; such effects can lead to the elevation of risk factors, and subsequently to an increase in T2DM risk. Gosadi *et al*.^13^ reported a statistically significant inverse association between inbreeding coefficients and age at diagnosis of T2DM, and further observed that such an association is stronger in patients where both parents were afflicted with T2DM. For reasons mentioned above, transfer of knowledge on T2DM genetic markers derived by global genome surveys (on Euro-centric populations) to the Arab population can be complicated by differences in disease prevalence, risk factor profiles, genetic risk allele frequencies, gene-environment interactions, and increased homozygosity for autosomal recessive genes.

In this study, we explore the genome-wide genotypes from a cohort of Arab individuals from Kuwait for gene loci associated with 13 metabolic traits - namely height, weight, waist circumference (WC), waist circumference to height ratio (WcHtR), body mass index (BMI), glycated hemoglobin (HbA1c), fasting plasma glucose (FPG), triglyceride (TGL), high density lipoprotein (HDL), low density lipoprotein (LDL), total cholesterol (TC), systolic blood pressure (SBP), and diastolic blood pressure (DBP). For this purpose, we choose Cardio-MetaboChip genotyping array, which has successfully been employed for fine-mapping of established loci in populations such as Han Chinese^14^ and Africans^15^. We scrutinize about 45,793 variants related to metabolic traits in 1,965 Arab ethnic individuals. We report a number of risk variants that are associated with metabolic traits at varying significant levels of p-values. Furthermore, we examine the broader context in which these genetic variants operate to alter metabolic disease states. The results of this study that map disease alleles in the indigenous Arab population can provide important perspectives on the pathogenesis and diagnosis of metabolic trait associated diseases.

## Results

### Sample and marker sets

Upon performing sample-related quality control steps (such as identification of duplicate samples [leading to removal of n = 1 sample], assessment of samples for call rates <97% [n = 232], assessment for gender mismatch [n = 74], identification of cryptic relatedness through IBD analysis [n = 151], and estimation of ancestry admixture to identify samples with ancestry mismatch [n = 39]), the sample size got reduced from 2,440 to 1,965. By performing marker-related quality control steps (such as MAF <0.01, HWE <0.001, genotype error <0.1, mind <0.1 [leading to removal of n = 68,582 markers], LD-pruning [n = 70,533], and considering only those markers that possess a minimum of 5% minor allele frequency in the study population [n = 24,740]), we narrowed down the number of MetaboChip markers from 196,725 to 45,793. We used these filtered sample and marker sets to perform association tests between markers and quantitative phenotype traits.

The scatter plot representing the first two principal components of the sample set is presented in Supplementary Figure S1. The plot depicts the three genetic substructures of Kuwaiti population that we established in our previous study^7^.

### Characteristics of study subjects

The demographic and biochemical characteristics of the study participants from the filtered set of 1,965 samples are shown in Table 1. The study population consisted largely of middle adulthood individuals (mean age = 44.88 ± 12.35 years) with an almost equal proportion of males to females (52%:48%). With a mean BMI of 31.53 ± 6.60 Kg/m^2^, the population could largely be categorized as Class I obese; the observed high mean value for waist circumference (at 100.82 ± 14.41 cm) further reflected the extent of obesity. As regards the disease status, 27% of the participants were diabetic, 26% were hypertensive, and 9% suffered from cardiovascular disorders. The mean value for fasting plasma glucose (FPG) measurements for the study population at 6.37 ± 2.90 mmol/l indicated prediabetes; the mean value for HbA1c at 6.28 ± 1.68 mmol/l indicated increased risk of diabetes; the mean values for LDL at 3.25 ± 0.095 mmol/l, HDL at 1.13 ± 0.035 mmol/l, triglyceride (TGL) at 1.58 ± 1.04* *mmol/l, and total cholesterol (TC) at 5.07 ± 1.07 mmol/l indicated normal or near optimal cholesterol levels. Mean values for systolic blood pressure (SBP) at 127.31 ± 17.06 mmHg and diastolic blood pressure (DBP) at 77.77 ± 10.73 mmHG indicated prehypertension.

### Markers associated with phenotype traits either at ‘close to genome-wide significant’ p-values or at ‘nominally significant’ p-values

Results from tests of association pointed to only one risk variant (namely Chr15:40531386-rs12440118 from *ZNF106*) associated with HbA1c emerging at ‘close to genome-wide significant’ p-value of 3.41E-08 (with Benjamini–Hochberg FDR p-value of 0.0032) (Table 2). Five other risk variants emerged as ‘nominally’ associated at the ‘lenient’ p-value threshold of 5.45E-07 (and Benjamini–Hochberg FDR p-value ≤ 0.05); and they were seen associated with FPG (rs7144734/[*OTX2-AS1,RPL3P3*]) and TGL (rs17501809/*PLGRKT*, rs11143005/*LOC105376072*, rs900543/[*THSD4,NR2E3*], and Chr12:101494770/*IGF1*) (Table 2). The markers continued to be associated at significant p-values when the models were adjusted for medication status (Table 2) or for diabetes status or for obesity status (Table 3) – e.g. under adjustment for obesity status, the p-values remained significant at ≤8.8E-07. All these 6 markers were identified by recessive models.

**Figure 1:**
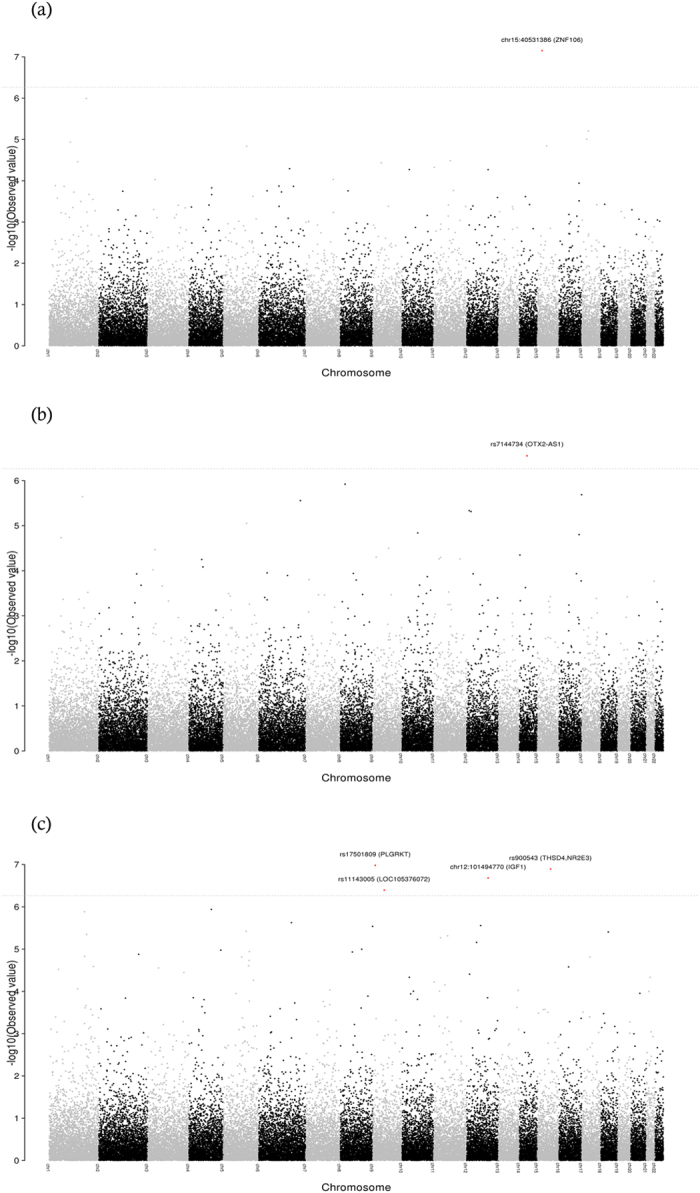
Manhattan plots of the 45793 tagSNPs employed to interrogate the genomes of the study population for associations with the phenotype traits of HbA1c (**1.a**), FPG (**1.b**), and TGL (**1.c**). The –log_10_ p-values were determined using linear regression methods adjusted for age, sex, first 10 principal components with respective quantitative trait. Labeled are those markers with a p-value of ≤ 5.45E-07.

The Manhattan plots of the interrogated SNPs for the phenotype traits of HbA1c, FPG, and TGL are presented in Fig. 1. The Quantile-Quantile plots are shown in Supplementary Figure S2. The genomic-control inflation factors (which compare observed association statistics against the expected distributions) corresponding to the 13 tested traits are presented in Supplementary Table S1; values of ≤1.04 were obtained for these factors and hence it was not felt necessary to perform corrections for genomic-control inflation on association statistics. Regional plots, depicting trait association statistics for all the SNPs (typed in the MetaboChip) from a region of 1MB, for the 6 identified markers are shown in Supplementary Figure S3.

Results from power calculations suggested that the sample size of 1965 used in this study has the potential to detect up to 2% quantitative trait variance with 80% power for the SNPs with MAF ≥5% at the claimed genome-wide significant p-value of 3.41E-08. The sample sizes for the different R^2^_G_ values are as follows (R^2^_G_, sample size): (0.015, 2677), (0.016, 2509), (0.017, 2360), (0.018, 2228), (0.019, 2109), (0.020, 2003), (0.021, 1907), (0.022, 1819), (0.023, 1739), (0.024, 1666), (0.025, 1598). The sample size of 1965 used in this study has the potential to pick up an effect size of <2.44 for HbA1c with the identified rs12440118 variant at 6% MAF (observed beta value = 2.00), <2.09 for FPG with the rs7144734 variant at 20% MAF (observed beta value = 1.465), <2.45 for TGL with the rs10860880 variant at MAF 6% (observed beta value = 1.59), <0.54 for TGL with the rs11143005 variant at 28% MAF (observed beta value = 0.4196), <1.625 for TGL with the rs900543 variant at 9% MAF (observed beta value = 1.625) at genome-wide significant p-value.

Upon searching the literature for evidences on the involvement of the identified gene loci (associated at either close to the stringent p-value threshold or at lenient p-value threshold) in metabolic trait related processes, the following affirmative observations were made:

#### Chr15:40531386 [*ZNF106*] with HbA1c

The variation leads to a non-synonymous amino acid change (W103R, aromatic to basic residue) in the encoded Zinc Finger Protein 106 (*ZNF106 alias ZFP106*). *ZNF106* is a product of *SIRM* gene and it shares the same initiation codon and differs in 3′ UTR with another product, namely *SH3BP3*, of *SIRM*^16^; thus *ZNF106* is expected to share the same properties of *SIRM*. The *SIRM* protein has been described as a novel insulin–regulated SH3 binding protein that associates with *Grb2* and *FYN*^17^. It is further known that deregulation of *Slc25a20* gene, which is known to play an important role in insulin secretion, is accompanied by the upregulation of *ZNF106* in Type 1 diabetes^18^. These evidences shown in mice and cell lines surmise that *ZNF106* could be a potential causal gene in human too and could be implicated in human insulin receptor signaling pathway.

#### rs7144734 [*OTX2-AS1,RPL3P3*] with FPG

*OTX2 antisense RNA 1 (head to head*) is a transcription factor known to play an important role in controlling expression of *OTX2* gene. Although there is no direct association of *OTX* with glucose metabolism or diabetes, it is known to be involved in regulating gonadotrophin releasing hormone (*GnRH*) in hypogonadism. Interestingly, a study from the Middle East region found that 36.5% of 1089 men with T2DM had low serum testosterone levels; 17% of such T2DM patients with low serum testosterone levels had primary hypogonadism while the remaining had secondary hypogonadism^19^. A study on *OTX2* knockout in mice confirms that *OTX2* is indispensable for *GnRH* expression^20^. The *GnRH* expression is found to be downregulated among T2DM men^21^.

#### rs17501809 [*PLGRKT*] with TGL

The Plasminogen receptor with a C-terminal lysine is a transmembrane protein, known to regulate catecholamine release^22^. Catecholamine is known to be involved in the regulation of lipoproteins including triglyceride metabolism^23^.

#### rs11143005 [*LOC105376072*] with TGL

This SNP is located in a long interspersed noncoding RNA (*lincRNA*) called *RP11-274B18.4-001* with no annotated function. In the gene expression data analysis (presented later in Results section), we find that this genotype upregulates the downstream *PGM5* gene which is known to be involved in glucose metabolism^24^. PGM activity is essential in the formation of glucose-6-phosphate from galactose and glycogen, and in the formation of carbohydrates from glucose-6-phosphate.

#### rs900543 [*THSD4,NR2E3*] with TGL

The nuclear receptor subfamily 2 group E member 3 (*NR2E3*) gene is implicated in several eye-related disorders including enhanced S-cone syndrome, a form of retinitis pigmentosa, and Goldmann-Favre syndrome. *NR2E3*, like other nuclear receptors, is a target for retinoic acid which is important in many physiological functions and in the expression of genes that regulate energy metabolism^25^. Till date there is no report on *NR2E3*’s direct role in lipid metabolism. However, discovery of *NR1D1* as an *NR2E3-*interacting protein^26^ adds a new dimension: *NR1D1* is known to regulate human *ApoC3* gene promoter, a gene that plays an important role in plasma triglyceride and remnant lipoprotein metabolism^27^.

#### Chr12:101494770 [*IGF1,PAH*] with TGL

Insulin-like growth factor 1 (*IGF1*) is similar to insulin in molecular structure. It plays an important role in cell growth and anabolism function. Recent study on *IGF1* deficient mice showed elevated levels of triglyceride^28^; and another study on *IGF1* treatment in healthy individuals showed reduced levels of fasting and postprandial triglyceride^29^. *IGF1* deficiency has been reported to be involved in the development of diabetic retinopathy in Thalassemia major patients from Italy^30^ and in the development of metabolic syndrome in a British cohort^31^.

### Additional susceptibility loci with suggestive evidence of association at p-values of <1.0E-05

It is also important to consider the markers that reached p-values of <1.0E-05 (‘suggestive’ evidence) in association tests. We identified a total of 30 such SNPs which were involved in 33 associations with 12 metabolic traits of height, weight, BMI, waist circumference (WC), waist circumference to height ratio (WcHtR), HbA1c, FPG, HDL, LDL, TGL, Total Cholesterol, and SBP (Table 4). 13 of these associations (involving 10 unique markers and 9 metabolic traits of weight, WC, WcHtR, BMI, HbA1c, LDL, HDL, TGL, and TC) were identified by models implementing additive mode of inheritance while 20 (involving 19 unique markers and 6 metabolic traits of weight, WC, HbA1c, FPG, TGL, and SBP) were identified by models implementing recessive mode of inheritance. All the associations identified under recessive mode of inheritance and 10 of the 13 associations identified under additive mode of inheritance were of risk effect.

For most of these suggestive associations, literature evidences for involvement of the gene loci in metabolic trait related processes were found (Table 5). The evidences are particularly prominent in the cases of [*TRA, TRD*] (WcHtR) and *C1orf106* (HbA1c), *GAPDHP56* (weight, BMI, and WC), *UST* (WC), [*MDGA1,ZFAND3*] (HbA1c), *CETP* (HDL), *BUD13* (TGL), *TMEM120B* (TGL), *TEX29* (weight), *KSR1* (HbA1c), *SLC28A3* (FPG), *PIK3C2G* (FPG), *LAMA4* (TGL), [*LY6D,GML*] (TGL), *MICAL2* (TGL), and *TTN* (SBP).

### Examining the EBI GWAS Catalog for reports on associations between the identified markers and the exact metabolic traits

By way of examining the GWAS Catalog (the NHGRI-EBI Catalog of published genome-wide association studies available at https://www.ebi.ac.uk/gwas/), we checked whether previous GWA studies report the associations identified in our study. None of the six markers, that we identified as associated in the study population (either at close to genome-wide significance or at nominal p-value threshold), was reported with significant associations in the EBI GWAS Catalog (Supplementary Table S2). However, two of the markers with suggestive evidence of association in our study (namely rs1800775/*CETP* with HDL, and rs9326246/*BUD13* with TGL) were seen associated with respective phenotype traits at genome-wide significant p-values in GWAS Catalog (see Supplementary Table S2). In all other instances, the p-values in the GWAS Catalog failed to reach genome-wide significance. Thus, it is quite possible that many of the associations identified in our study are novel. We noticed that the effect sizes were not consistent between the data from Arab ethnicity presented in this study and the publicly available results in GWAS Catalog (except in the case of rs9326246/*BUD13* marker with beta values of 0.237 versus 0.2185). In most instances of identified associations, the beta values were close to 0 (ranging from −0.01 to + 0.01) in GWAS Catalog; as studies in the GWAS Catalog were carried out with large number of samples, it is not surprising to find values close to 0 for the effect size. However, it is to be noted that the effect sizes are not very informative when the p-values are not significant.

### Examining the EBI GWAS Catalog for reports on associations between the identified markers and related (not necessarily the exact) metabolic traits

Upon examining further the EBI GWAS Catalog for associations between the markers and related (not necessarily the exact associated trait in our study population) metabolic traits in other populations (see Online Supplement Data 2); we found that 17 of the 36 identified markers showed associations with related metabolic traits at p-values of ≤E-03 (highlighted in blue in Online Supplement Data 2). In the case of four of these markers, identified in our study as associated at nominal p-values (rs900543 with TGL, rs11143005 with TGL) or at suggestive evidence of p-values (rs17569297 with TGL, and rs10935794 with total cholesterol), associations at p-values of <E-04 were reported in other populations with related metabolic traits (rs900543 with fasting insulin at p-value = 9.40E-05, rs11143005 with 2 hour fasting glucose at p-value = 4.47E-05, rs17569297 with HDL at p-value = 1.51E-06, and rs10935794 with serum ratio of arabinosefructose at p-value = 9.840E-05) (Supplementary Table S3). Particularly interesting was the rs17569297 marker that showed a p-value at suggestive evidence of association in both our study population and the European population.

### Performance of most replicated exemplary markers relating to obesity and diabetes in our study population

Established susceptibility gene loci for traits relating to obesity and diabetes in European population have been tested for replication in other ethnic population groups; such exemplary loci that are known to replicate well in other ethnic population groups (such as Indians and Russians) include *PPARg, KCNJ11, TCF7L2, SLC30A, ABCC8, HHEX, CDKN2A, IGF2BP2, CDKAL1,* and *FTO*^32^,^33^,^34^. We examined the performance of markers from these gene loci in our MetaboChip data set (Supplementary Table S4). None of the markers from *ABCC8, HHEX, CDKN2A, IGF2BP2,* and *FTO* surfaced with at least a p-value of ≤0.05 for association in our data set. Markers from the other genes *PPARg, KCNJ11, TCF7L2, SLC30A,* and *CDKAL1* did not reach significant association with any of the 13 tested metabolic traits; at best it reached a p-value of 0.001.

### Gene expression regulation by identified markers

Examination of the Genotype-Tissue Expression (GTEx) data showed that seven of the 36 identified markers differentially regulate the expression of either the downstream or upstream genes (Table 6); such markers are Chr15:40531386 [*ZNF106*; W->R], rs11143005 [*LOC105376072*], rs1800775 [*CETP*], rs9326246 [*BUD13*], rs17716285 [*KSR1*], rs11777524 [*LY6D, GML*], and rs10497520 [*TTN*; K->E]. In addition, the rs11143005 marker from the above list was seen as regulating its own gene. It is interesting to note that in four instances the regulated up/downstream genes were also related to metabolic traits. Such instances are listed below:

#### rs11143005 [*LOC105376072*] with TGL

This SNP is located in a long interspersed noncoding RNA (*lincRNA*) called *RP11-274B18.4-001* of unknown function. As per GTEx data, this gene upregulates its downstream gene *PGM5* (phosphoglucomutase 5), which is involved in glucose metabolism^24^. *PGM* activity is essential in the formation of carbohydrates from glucose-6-phosphate, and in the formation of glucose-6-phosphate from galactose and glycogen.

#### rs17716285 [*KSR1*] with HbA1c

The rs17716285 SNP from *KSR1* upregulates the expression of an upstream gene *NOS2* (Nitric oxide synthase 2). It has been strongly suggested that nitric oxide plays a crucial role in the regulation of blood pressure^35^,^36^.

#### rs1800775 [*CETP*] with HDL

The rs1800775 SNP from *CETP* downregulates the upstream *NLRC5* gene. Charlesworth *et al*., through combining results from association tests with genome-wide transcriptional profiling data for HDL, identified *NLRC5* as a novel loci associated with HDL^37^.

#### rs10497520 [*TTN*] with SBP

The rs10497520 SNP from *TTN* gene downregulates the upstream *FKBP7* gene. Peter *et al*. report that the *FKBP7* gene showed a significant association with intensive lifestyle intervention response among overweight/obese diabetic individuals not receiving beta-blocker medications^38^.

### Pathway Analysis

In order to identify the pathways that are enriched with the reported gene loci, we mapped the genes onto canonical pathways. We found that five of the identified gene loci (*KSR1, PIK3C2G, MICAL2, CETP,* and *UST*) are involved in the following metabolic pathways: Ceramide signaling (*KSR1* and *PIK3C2G;* p-value for overlap = 9.56E-04), pregnenolone biosynthesis (*MICAL2*; p-value for overlap = 4.07E-03), ERK/MAPK signaling (*KSR1* and *PIK3C2G*; p-value for overlap = 5.08E-03), Histidine Degradation VI (*MICAL2*; p-value for overlap = 5.08E-03), and *LPS/IL-1* mediated inhibition of *RXR* function (*CETP* and *UST*; p-value for overlap = 6.97E-03). As enumerated below, these pathways relate to processes and disorders relating to metabolic traits:

#### Ceramide Signaling

This signaling pathway is implicated in the pathogenesis of insulin resistance and other obesity-associated metabolic diseases^39^.

#### Pregnenolone biosynthesis

Pregnenolone is synthesized from cholesterol and is involved in causing obesity and insulin resistance^40^.

#### ERK/MAPK signaling

This pathway is one of the well-studied pathways in the context of metabolic disorders. Cross-talk between the epidermal growth factor receptor (*EGFR*)–activated *MAPK/ERK* pathway and insulin signaling pathway in controlling glucose metabolism has been demonstrated in Drosophila^41^.

#### Histidine Degradation VI

It is well known that dysregulation of insulin leads to marked alteration in amino acid metabolism, and that histidine supplementation improves insulin resistance and reduces obesity^42^.

#### LPS/IL-1 Mediated Inhibition of RXR function

This pathway is known to be involved in causing diet-induced obesity, and noninsulin-dependent diabetes mellitus^43^.

**Figure 2:**
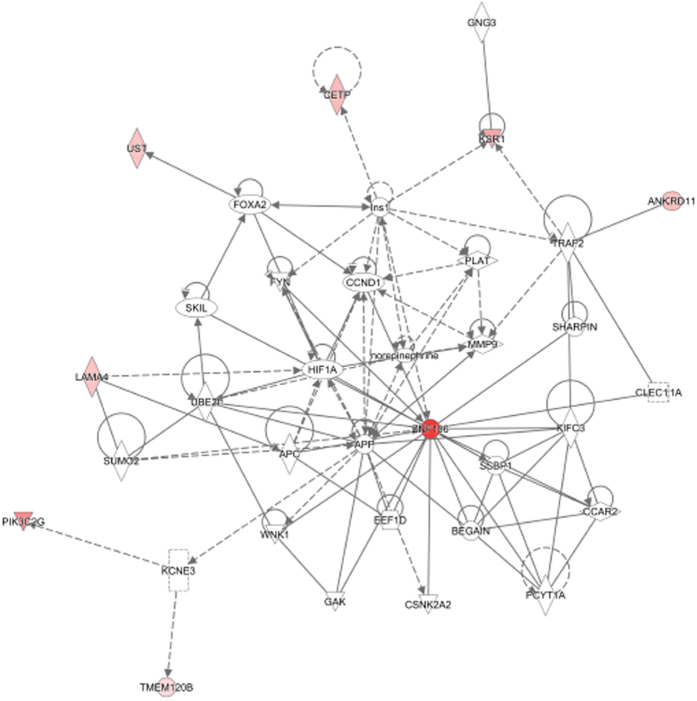
Top ranked gene network interacting with gene loci identified in our study. The genes from our study are shown in red.

Figure 2 depicts gene interaction network showing highest overlapping score. The identified gene loci (namely *UST, CETP, MEM120B, ZNF106, KSR1, ANKRD11, LAMA4,* and *PIK3C2G*), that overlap with the above-mentioned pathways, were all seen interacting with one another (shown in red color) in the interaction network. The other interacting partner genes were also known to be involved in the etiology of complex metabolic disorders. For example: *PLG, PLAT, PLAUR, norepinephrine, TNF, MMP9, FYN* and *SHARPIN* are involved in the etiology of dyslipidemia, hypertension, and cardiovascular disease (CVD); *APC* and *CCND1* are associated with obesity; *SUMO2, SMAD2, INS1, WNT1, TGFB1, TRAF2,* and *CCAR2* are associated with diabetes; whereas *CCND1, TNF, MMP9*, and *norepinephrine* are associated with comorbid conditions of metabolic disorders.

### Transethnic variation in allele frequencies

Frequencies of risk alleles for T2DM and metabolic traits often vary between populations, and thereby cause population differences in the risk due to particular genetic factors. The identified thirty six markers are common markers (*i.e.* MAF >5%) in the study population. Examination of allele frequencies at these markers in 1000 Genomes Project Phase 3populations (Table 7) revealed that as many as twenty four out of thirty six markers appear as low-frequency variants (MAF <5%) in one or more global populations; and in as many as half of the instances, they appear as rare variants (at MAF< =1%) in East Asian (15 times) and/or African (11 times) populations. Upon considering the 36 markers to establish Pearson correlation coefficients, we found that the risk variants from Arab population showed least correlation with East Asian populations at 0.412 [p-value = 0.00737, CI = 0.12 to 0.63] and highest correlation with South Asians at 0.738 [p-value = 3.604E-08, CI = 0.55 to 0.85]. The values for correlation coefficients with populations from the 1000 Genomes Project Phase 3 were intermediate, ranging from 0.588 to 0.647 (with AMR - Ad Mixed Americans, 0.588 [p-value = 5.19E-05; CI = 0.34–0.75], with EUR – Europeans, 0.647 [p-value = 4.84–06; CI = 0.42–0.79], with AFR – Africans, 0.654 [p-value = 3.43E-06; CI = 0.43–0.80]). These observations illustrate transethnic variations in the penetrance of risk alleles.

## Discussion

Overall, this study identified (Chr15:40531386-rs12440118/*ZNF106*/W->R) as a risk variant associated with HbA1c at close to genome-wide significant p-value, and five other risk variants ‘nominally’ associated (p-value ≤ 5.45E-07) with fasting plasma glucose (rs7144734/[*OTX2-AS1,RPL3P3*]) and triglyceride (rs17501809/*PLGRKT*, rs11143005/*LOC105376072*, rs900543/[*THSD4,NR2E3*], and Chr12:101494770/*IGF1*). Involvement of these gene loci in metabolic traits was amply supported by literature evidences. Furthermore, the study identified 30 additional variants with suggestive evidence of association with the phenotype traits of height, weight, BMI, waist circumference (WC), waist circumference to height ratio (WcHtR), HbA1c, FPG, HDL, LDL, TGL, total cholesterol, and SBP. Most of these gene loci with suggestive evidence of association - particularly [*TRA, TRD*] (WcHtR), *C1orf106* (HbA1c), *GAPDHP56* (weight/BMI/WC), *UST* (WC), [*MDGA1,ZFAND3*] (HbA1c), *CETP* (HDL), *BUD13* (TGL), *TMEM120B* (TGL), *TEX29* (Weight), *KSR1* (HbA1c), *SLC28A3* (FPG), *PIK3C2G* (FPG), *LAMA4* (TGL), [*LY6D,GML*] (TGL), *MICAL2* (TGL), and *TTN* (SBP) - were supported by literature evidences on their involvement in metabolic trait related processes.

Most of the reported variants are harbored in either intronic or intergenic regions. However, two of the identified markers lead to non-synonymous amino acid changes in the encoded proteins; these missense variants are: Chr15:40531386/*ZNF106*/W->R associated with HbA1c at close to genome-wide significant p-value, and rs10497520/*TTN*/K->E with suggestive evidence of association (p-value < 1.0E-05) with SBP. In both these instances, the amino acid substitution leads to change in the charge associated with the side chain. In the case of W->R (*trp*-> *arg*) substitution, while both the amino acids are hydrophobic, *trp* has non-charged side chain and *arg* has positively charged side chain. In the case of K->E (*lys*->*glu*), while both the amino acids are hydrophilic, *lys* is positively charged and *glu* is negatively charged. Thus, though the tendency to get buried into protein core or to get exposed on the surface is not changed, the polarity is altered. Change in polarity may affect the interactions that the amino acid residue has with other residues from the protein or with non-protein molecules.

We find it striking that all the 6 associations identified either at close to genome-wide significance or at the lenient p-value threshold of ≤5.45E-07, and as many as 20 of the 33 associations identified with suggestive evidence at p-values of <1.0E-05 operate under recessive mode of inheritance and have risk effect (Tables 2–3). Associations with protective effect were seen only in four cases (involving three unique variants), all operating under additive mode of inheritance. Hence, our results suggest that recessive models in addition to additive models are to be used to study genetics of metabolic traits in Arab population.

It is distinctly seen that some of the identified gene loci are also associated with genetic disorders that are prevalent in the Arabian Peninsula - such gene loci include: *C1orf106* associated with autoimmune disorders; [*OTX2-AS1,RPL3P3*] with hypogonadism; [*THSD4,NR2E3*] in several eye-related disorders including enhanced S-cone syndrome; [*IGF1,PAH*] in diabetic retinopathy, growth retardation with deafness and mental retardation (due to *IGF1* deficiency); [*TRA,TRD*] in immune-related disorders; *LAMA4* in diabetic nephropathy; and *KSR1* in diabetic vascular complications. Some of these disorders (such as S-cone syndrome, and growth retardation with deafness and mental retardation due to *IGF1* deficiency) follow autosomal recessive inheritance. Pleiotropic effect of recessive genetic signatures on complex disorders cannot be ignored. Many studies report coexistence of recessive diseases with complex disorders in specific populations - interesting examples include: (i) Celiac with Type 1 diabetes (T1DM): Prevalence of celiac among T1DM patients was seen as 8.3%^44^ in an Iranian cohort and as 11.3% in a Saudi Arabian cohort^45^; (ii) Hypogonadism with T2DM: prevalence of Hypogonadism in T2DM patients was seen as 24.3% in a Jordanian cohort^46^, and 17.3% in an Iranian cohort^47^; and (iii) Pigmentosa, hypogonadism and T2DM: A study from Saudi Arabia reported at least 2 cases with coexistence of retinitis pigmentosa, hypogonadism and T2DM^48^. Nevertheless, very limited information is available on the nexus of molecular cascades involving the role of consanguinity and recessive genes in complex disorders.

Involvement of some of the identified gene loci (namely Chr15:40531386/*ZNF106*, Chr12:101494770/[*IGF1,PAH*], rs883431/*SLC28A3*, rs925530/*TMEM120B*, and rs17716285/*KSR1*) in processes relating to metabolic traits have been previously demonstrated in mice or cell lines^18^,^28^,^49^,^50^,^51^ (see Table 5). Therefore, our study gives suggestive evidence for involvement of these gene loci in human too.

Further, associations of markers from some of the identified gene loci with metabolic traits have been demonstrated in other population groups (see Table 5); such loci include:

1. rs10005556/*GAPDHP56* - genome region in *GAPDHP56* is associated with total fat mass in UK10K cohort^52^
2. rs1184476/*TEX29* - variants of *TEX29* are associated with age at menarche among Chinese women^53^
3. rs17639988/[*MDGA1,ZFAND3*] - SNPs that are intergenic between *MDGA1* and *ZFAND3* are implicated in T2DM in East Asian population^54^
4. rs4764409/*PIK3C2G* - Variants from this gene are found to be associated with high HbA1c and low serum insulin levels in Japanese T2D population^55^.

Furthermore, in the following two cases of identified suggestive markers, significant associations have been previously established with the respective metabolic traits in Euro-centric populations (Supplementary Table S2):

1. rs1800775/*CETP*/HDL - the EBI GWAS Catalog reports this variant as significantly associated with HDL in European population (with a p-value of ≤3.33E-644) and in mixed populations (with a p-value of ≤2.05E-306)^56^,^57^.
2. rs9326246/*BUD13*/TGL - the EBI GWAS Catalog reports this variant as significantly associated with TGL in European population (with a p-value of ≤1.27E-229), in populations included in the CARDIoGRAM consortium (with a p-value of ≤4.70E-124) and in mixed populations (with a p-value of ≤4.79E-124)^56^,^57^,^58^,^59^.

In addition, the rs17569297 identified in our study as associated with TGL at suggestive evidence of p-value has been reported as associated with a related metabolic trait of HDL in European population at similar p-values^60^ (Supplementary Table S3).

It is revealed that seven of the identified genetic variants lead to differential expression of either the downstream or upstream genes (see Table 6). Interestingly, four of these seven genes (namely *PGM5* regulated by rs11143005, *NLRC5* by rs1800775, *NOS2* by rs17716285, *FKBP7* by rs10497520) have literature evidence for associations with metabolic traits^24^,^35^,^36^,^37^,^38^ and thus these genes are potential candidate genes for further functional studies on processes relating to metabolic traits in this population.

The identified gene loci was seen to map to the canonical pathways of Ceramide signaling, Pregnenolone biosynthesis, *ERK/MAPK* signaling, Histidine Degradation, and *LPS/IL-1* Mediated inhibition of *RXR* function; all these five pathways relate to pathogenesis of obesity, insulin resistance and signaling. Key gene loci (apart from the loci identified in this study) that mapped to these pathways include *KSR1, PIK3C2G, MICAL2, CETP, UST, TMEM120B, ZNF106, ANKRD11,* and *LAMA4*. Gene interaction network analysis revealed extensive interactions between these genes and others that are involved in etiology of dyslipidemia/hypertension/CVD (such as *PLG, PLAT, PLAUR, norepinephrine, TNF, MMP9, FYN* and *SHARPIN*), obesity (such as *APC* and *CCND1*), T2DM (such as; *SUMO2, SMAD2, INS1, WNT1, TGFB1, TRAF2,* and *CCAR2*), and comorbid conditions of metabolic disorders (such as *CCND1, TNF, MMP9*, and *norepinephrine*). All these interacting genes warrant future targeted genotyping studies for metabolic traits in Arab population.

Upon viewing the results from pathway analysis and gene expression (GTEx) data analysis in the context of our previous studies on leptin-mediated hypertension^61^,^62^, we can hypothesize that ceramide signaling is probably a key pathway connecting obesity-induced diabetes and obesity-induced hypertension in Arab population. Ceramide signaling plays a role in central control of feeding via regulating leptin levels^63^. Two of the identified gene loci in our study namely *KSR1* and *PIK3C2G* (associated with HbA1c and FPG, respectively) are mapped to this pathway. Interestingly, in addition to this, the presented GTEx results revealed that rs17716285 harbored in *KSR1* upregulates its upstream *NOS2* (Nitric oxide synthase 2) gene. Experimental studies have shown that glucose releases endothelial nitric oxide (NO), which in turn contributes to renal hyperperfusion in models of diabetes; upon examining whether this translates into the human condition, Schneider *et al*.^64^ reported that poor glycaemic control is related to higher NO activity which in turn leads to hypertension. Plenty of research reports exist suggesting that normal release of nitric oxide plays a crucial role in homeostasis of blood pressure, and that impairment of NO level can cause hypertension^35^,^36^. However, models that implement the role of NO in the pathogenesis of the comorbidity (of diabetes and hypertension) are yet to be demonstrated. The model (incorporating the genes from the ceramide signaling) that we propose in this study requires further functional studies in this population.

Studies to identify genetic markers for the metabolic traits of HbA1c and FPG are usually performed on healthy individuals^65^. Efforts to replicate the identified markers/loci in individuals with diabetes have not resulted in much success; it is yet to be established whether the low reproducibility is due to differences in study power or in ethnicity, or in environment (such as medication for diabetes)^65^. The Kuwaiti population, which contributed to our study cohort, has a high prevalence of obesity and diabetes^2^,^66^; as a result, 54% of our study participants are obese, 27% are diabetic, and 13% are under diabetes medication (Table 1). Upon introducing adjustment for diabetes status or for obesity in association tests, the p-values and beta values still remained significant (Table 3). In our future studies, we will focus on replicating these markers in entirely non-diabetic/non-obese cohort and in entirely diabetic/obese cohort.

A potential limitation of this study relates to absence of an independent sample set for replicating the results. However, as discussed so far, most of the identified associations benefit from literature evidence - involvement of some of the identified gene loci in metabolic processes have been demonstrated in mice or cell lines; markers from some of the identified gene loci have been established to be associated with metabolic traits in previous GWA studies; and two of the markers (rs1800775 [from *CETP* gene and associated with HDL]; rs9326246 [from *BUD13* gene and associated with TGL]) with suggestive evidence of association have been reported in other European populations as associated with the respective traits at genome-wide significant p-values. In spite of the absence of replication using an independent sample set, the study presents to the community valuable data on potential markers associated with metabolic traits in an ethnic population which has been under-represented in global genome-wide surveys.

Our study could identify only one marker at a p-value close to genome-wide significance; this might be because of small sample size. A mention about limitations in achieving a large target for participant recruitment is in order here. Establishment of Arab ethnicity as part of the recruitment process was rigorous - ethnicity of every participant was confirmed via a rigorous questionnaire that addressed parental lineages up to three generations. Participants, selected from the computerized register of the Public Authority of Civil Information (PACI) by way of random sampling, were invited to visit us if they wished to participate in the research project; the acceptance rate by the participants was low as there exists a cultural barrier for the local Arabs to disclose their tribe ancestry and to donate blood samples. In spite of the achieved small sample size, the reported loci are promising and they deserve to be examined in large-sized Arab cohorts.

In conclusion, results based on our study with Arab population from Kuwait pinpoint crucial genes that take part in metabolism and metabolic disorders. The presented Pathway analysis and Genotype Tissue Expression data analysis reveal the broader context in which genetic variants regulate metabolic processes and suggests further gene loci for future studies on metabolic traits in Arab population. The study also demonstrates the association of recessive signatures and their pleiotropic effects with the etiology of metabolic disorder. Culturally and geographically distinct ethnic populations such as those of Arabian Peninsula are under-represented in global genome survey studies; the presented study bridges this gap.

## Methods

### Study participants

A total of 2,440 participants from Kuwait were randomly recruited under protocols approved by the scientific and ethics advisory boards at Dasman Diabetes Institute. The participants include: **(i)** A random representative sample of adults (>18 years of age) of Arab ethnicity across the six governorates of the State of Kuwait. A stratified random sampling technique was used for the selection of participants from the computerized register of the Public Authority of Civil Information (PACI). PACI is a government body which maintains all records of personal information of both Kuwaiti citizens as well as expatriates (that includes citizens of other Arab countries from the region); and **(ii)** Patients with diabetes or prediabetes seeking tertiary medical care in clinics at our institute. At the time of participant recruitment, the nationality was confirmed and ethnicity of every participant was confirmed via a rigorous questionnaire that addressed parental lineages up to three generations; data on age, sex, illness (e.g. diabetes and cardiovascular complications), and medication were recorded; and furthermore, vital signs such as height, weight, waist circumference (WC), and blood pressure readings were recorded. Informed consent was obtained from each of the participants. Participant recruitment for this study was carried out as part of approved research projects^7^,^66^ at Dasman Diabetes Institute.

### Sample collection and genotyping

Upon signing written consent forms, blood samples were collected after confirming that the participants were fasting overnight. The methods to collect blood samples and to measure vital signs were carried out in accordance with guidelines laid in place by the institutional Ethical Review Committee. Lipid profile and glucose measurements were recorded. Gentra Puregene^®^ kit (Qiagen, Valencia, CA, USA) was used to extract DNA as per manufacturer’s protocols. DNA was quantified, with the requirement that the A260/A280 ratio is in the range of 1.8–2.1, using both Quant-iT™ PicoGreen^®^ dsDNA Assay Kit (Life Technologies, NY, USA) and Epoch Microplate Spectrophotometer. Frozen DNA stocks were diluted to a working solution of concentration at 50 ng/μl as recommended by Illumina (Illumina, CA, USA). Samples were genotyped using the Illumina HumanCardio-MetaboChip array utilizing Infinium HD Assay Ultra genotyping assay methods. Genotyping assay includes whole genome amplification, fragmentation, hybridization, staining and imaging of Cardio-MetaboChips using the Illumina iSCAN system. We distributed the 2,440 samples onto 27 batches (each homogenized for sex and diabetes status) and then carried out genotyping. The MetaboChip is a custom genotyping array based on the Illumina iSelect platform and is designed for use in replication of top association signals from the largest available GWAS meta-analysis for metabolic-related traits; it fine mapped 257 genome-wide significant association signals for 15 of these traits^67^. The MetaboChip contains ~200,000 SNP markers that include rare variants identified in the 1000 Genomes Project as well.

### Power calculation

We performed power calculation using Quanto software version 1.2.4 (University of Southern California, http://biostats.usc.edu/Quanto.html). Power calculation was performed separately for additive and recessive genetic models. We considered ‘Gene only’ hypothesis in independent individuals with quantitative traits. Marginal genetic effect estimate (R_G_^2^) was set to range from 0.015 to 0.025 by incrementing by 0.001 (so as to detect a genetic effect that can explain at least 1.5% to 2.5% of the trait variance). For each of the phenotype traits, population mean ± SD value of the respective quantitative trait (Table 1) was used. Power for the analysis was set at 80% and type 1 error at genome-wide significant p-value was considered.

### Quality control and statistical analysis

Raw intensity data from all of the 27 batches were pooled and genotypes were called using GenCall algorithm implemented in GenomeStudio software. Samples with call rates <97% were removed. A high quality set of SNPs was derived by applying a series of quality metric thresholds: SNPs with call rate of <98%, of low intensity (AB R Mean ≤0.25), poor cluster separation (Cluster Sep <0.3), with heterozygote clusters too close to homozygotes (AB T Mean ≤0.2 or ≥0.8), excess of heterozygotes (Het Excess ≥0.2), and those with fewer than expected heterozygotes (Het Excess ≤−0.3) were removed. We used GenomeStudio software for gender estimation and removed those samples reporting gender mismatches. We also excluded duplicate samples.

We used PLINK^68^ to perform quality control procedures on markers. Markers that failed to reach cut-off values for missingness per individual (–mind 0.1), allele frequency (–MAF 0.01), missingness per marker (–geno 0.1), and Hardy-Weinberg equilibrium (HWE < 10^−3^) were ignored. These procedures reduced the size of marker set to 128,143 SNPs. Since the markers mapped in MetaboChip are from gene loci known to be associated with metabolic disorders and related traits, the association tests would pick up a high number of markers that are in linkage disequilibrium (LD) with one another. Hence, we pruned LD with ‘–indep-pairwise’ option in PLINK with an R^2^ value > 0.3 with any other SNP within a 50-SNP sliding window (advanced by 5 SNPs each time); this stringent LD pruning step ensures that no two SNPs are in strong LD with one another.

We examined the sample set for relatedness using PLINK’s ‘–genome’ feature (PI_HAT > 0.125, *i.e.* up to third degree relatives) and randomly removed one sample per pair of related individuals. We used ADMIXTURE software^69^ for ancestry estimation and excluded samples with improper ethnicity.

We used EIGENSTRAT^70^ for performing Principal component (PC) analysis with the following choices for parameters: number of eigenvectors to output (numoutevec = 10), turn off outlier removal (numoutlieriter = 0), number of principal components along which to remove outliers during each iteration (numoutlierevec = 10), and number of standard deviations which an individual must exceed along one of the top (numoutlierevec) principal components in order for that individual to be removed/classified as an outlier (outliersigmathresh = 6.0). All the 10 principal components were used as covariates in quantitative trait association tests to adjust for population stratification.

Quantitative trait association analysis was performed, using linear regression method available in PLINK, for each of the 13 traits namely height, weight, waist circumference (WC), waist circumference to height ratio (WcHtR), body mass index (BMI), glycated hemoglobin (HbA1c), fasting plasma glucose (FPG), triglyceride (TGL), high density lipoprotein (HDL), low density lipoprotein (LDL), total cholesterol (TC), systolic blood pressure (SBP), and diastolic blood pressure (DBP). Additive mode of inheritance is the most common method used in global GWA studies. However, considering the genetic background of this population, we also used models based on recessive mode of inheritance. Tests of association were adjusted for age, sex, and the first 10 principal components (denoted as R, Regular Adjustments). The tests of associations were repeated with further adjustment for medication status (denoted as R + M, regular and medication adjustments) - all the anthropometric and lipid traits were adjusted for lipid lowering medication; FPG and HbA1c were adjusted for diabetes medication and hypertension traits were adjusted for hypertension medication.

### Regional, Q-Q, Manhattan and PCA plots

In order to generate regional association plot for a SNP-trait association, all the SNPs (typed in MetaboChip) from a region of around 1MB centered on the SNP were tested for association with the trait; the resultant statistics and the SNPs were displayed in the regional association plot. Region-plot tool (https://github.com/pgxcentre/region-plot) was used to produce regional plots. Q-Q plots, Manhattan plots and PCA plots were generated using R scripts (https://cran.r-project.org).

### P-value thresholds for assessing the statistical significance of genotype-phenotype associations

#### P-value threshold for association at genome-wide significance

The p-value threshold for genome-wide significance needs to be calibrated for the 13 tested traits, for the 2 tests pertaining to adjustments for medication status, and for the two genetic models of recessive and additive modes of inheritance; the p-value threshold derived using such a stringent criteria is 2.10E-08 (=0.05/[45,793*2*13*2]). However, the 13 traits are probably correlated to some extent and hence the effective number of independent traits can be lesser than 13. Upon performing Spearman correlation analysis between the 13 traits and then calculating effective number of independent traits using matSpD tool^71^ (http://neurogenetics.qimrberghofer.edu.au/matSpD/), it turned out that the effective number of independent traits is 8.04. By way of considering the number of markers tested, number of genetics models, number of tests considering medication correction, and effective number of independent traits, we found 3.41E-08 (=0.05/[45793*2*8*2]) as the ‘stringent’ p-value threshold for genome-wide significance. We also required that the associations passed through Bonferroni correction and Benjamini–Hochberg FDR correction procedures at p-values ≤ 0.05.

#### P-value threshold for association at nominal significance

We further defined a ‘lenient’ p-value threshold of 5.45E-07 (=0.05/[45,793*2]) calculated on the basis that the tests for multiple traits are independent and are not concurrent. We termed the associations whose p-values passed through such a lenient p-value threshold as ‘nominal’ associations.

#### P-value threshold for associations with suggestive level of significance

We further defined a third category of markers as showing “suggestive evidence of association” if the observed p-values were < 1.0E-05.

### Expression data, pathway, and allele frequency analysis

To get insights into the manifestation of the effects that the identified variants might exert on regulation of gene expression in human tissues, we examined the database of expression quantitative trait loci using the GTEx (Genotype-Tissue Expression) portal available at http://www.gtexportal.org. Pathway analysis for the identified gene loci from this study was performed using Ingenuity Pathway Analysis software (IPA, QIAGEN Redwood City, www.qiagen.com/ingenuity). Transethnic frequency analysis on the identified markers was performed by correlating 1000 Genomes Project Phase 3 allele frequencies for the African (AFR), Ad Mixed American (AMR), East Asian (EAS), European (EUR), and South Asian (SAS) populations to those in the study population using Pearson-correlation tests.

## Additional Information

**How to cite this article**: Hebbar, P. *et al*. Genetic risk variants for metabolic traits in Arab populations. *Sci. Rep.* **7**, 40988; doi: 10.1038/srep40988 (2017).

**Publisher's note:** Springer Nature remains neutral with regard to jurisdictional claims in published maps and institutional affiliations.

## Supplementary Material

Supplementary Information

Supplementary Dataset 1

## Figures and Tables

**Table 1:** The demographic and biochemical characteristics of the study participants.

Characteristics	Study Participants
Sex (M:F)	1028:937
Age (*in years* ± *SD*)	44.88 ± 12.35
Height (*cm* ± *SD*)	165.74 ± 9.20
Weight (*Kg* ± *SD*)	86.59 ± 19.24
BMI (*Kg/m*^*2*^ ± *SD*)	31.53 ± 6.60
Waist circumference,WC (*cm* ± *SD*)	100.82 ± 14.41
Waist circumference to Height ratio, WcHtR (±*SD*)	0.602 ± 0.111
Fasting plasma glucose, FPG (*mmol/l* ± *SD*)	6.37 ± 2.90
Glycated hemoglobin, HbA1c (*mmol/l* ± *SD*)	6.28 ± 1.68
Low-density lipoprotein, LDL (*mmol/l* ± *SD*)	3.25 ± 0.95
High-density lipoprotein, HDL (*mmol/l* ± *SD*)	1.13 ± 0.35
Triglyceride, TGL (*mmol/l* ± *SD*)	1.58 ± 1.04
Total Cholesterol,TC (*mmol/l* ± *SD*)	5.07 ± 1.07
Systolic blood pressure, SBP (*mmHg* ± *SD*)	127.31 ± 17.06
Diastolic blood pressure, DBP (*mmHg* ± *SD*)	77.77 ± 10.73
Obesity (Class I and more – i.e. BMI> = 30 Kg/M^2^) (*case:control*)	1062:903
Type 2 Diabetes (*case:control*)	536:1429
Hypertension (*case:control*)	513:1452
Cardiovascular disease, CVD (*case:control*)	185:1780
Lipid lowering medication@ (*Yes:No*)	103:1862
Diabetes medication@ (*Yes:No*)	249:1716
Hypertension medication@ (*Yes:No*)	230:1735

^@^It reflects whether medication is administered around the time of participant registration; previous records of medication were not checked.

**Table 2:** Markers that are shown to be associated with phenotype traits either at close to the claimed genome-wide significant p-value of ≤3.41 × 10^−08^ or at nominally significant p-value of ≤5.45 × 10^−07^.

Phenotype	Variant	Gene	Model	MAF	Beta$	P-value$	Beta#	P-value#	Bonferroni p-value	Benjamini–Hochberg FDR p-value
**A. Association at close to genome-wide significance of p-value ≤ 3.41E-08**										
HbA1c	chr15:40531386-rs12440118	*ZNF106* (W > R)	Recessive	0.1059	2.006	7.07E-08	1.498	2.70E-05	0.003	0.0032
**B. Associations at nominal significance of p-value ≤ 5.45E-07**										
	rs7144734	*OTX2-AS1* (upstream)	Recessive	0.2036	1.465	2.82E-07	1.361	4.31E-07	0.012	0.012
TGL	rs17501809	*PLGRKT* (intronic)	Recessive	0.0593	1.807	1.04E-07	1.74	3.29E-07	0.0047	0.0016
TGL	rs11143005	*LOC105376072* (intronic)	Recessive	0.2829	0.4196	4.03E-07	0.4225	3.21E-07	0.018	0.0030
TGL	chr12:101494770/rs10860880	*IGF1* (downstream)	Recessive	0.05575	1.596	2.077–07	1.603	1.74E-07	0.0095	0.0019
TGL	rs900543	*THSD4, NR2E3* (intergenic/downstream to *NR2E3*)	Recessive	0.08696	1.625	1.27E-07	1.607	1.67E-07	0.0058	0.0016

^$^Tests for association were adjusted for age, sex, and the first 10 principal components.

^#^Tests for association with anthropometric and lipid traits were adjusted for age, sex, principal components, and lipid lowering medication status; tests for association with HbA1c and FGL were adjusted for age, sex, principal components, and diabetes medication status; tests for association with SBP and DBP were adjusted for age, sex, principal components, and hypertension medication status.

**Table 3:** Effect of adjusting association tests for diabetes status and obesity status with markers identified at either close to the claimed genome-wide significant p-value of ≤3.41E-08 or at nominally significant p-value of ≤5.45E-07.

Marker and gene loci@	Metabolic trait	Statistics after regular adjustment (RA) for age, sex, and the first 10 principal components and further adjustments for Diabetes medication (DM), Diabetes status (DS), and BMI as a surrogate for Obesity Status (OB)							
		RA		RA + DM		RA + DS		RA + OB	
		Beta	p-value	Beta	p-value	Beta	p-value	Beta	p-value
chr15:40531386-rs12440118 *ZNF106* (W > R)	HbA1c	2.006	7.07E-08	1.50	2.705E-05	1.55	9.30E-05	1.96	1.17E-07
rs7144734 *OTX2-AS1* (upstream)	FPG	1.465	2.825E-07	1.36	4.312E-07	1.12	2.18E-06	1.51	8.8E-07
rs17501809 *PLGRKT* (intronic)	TGL	1.807	1.043E-07	1.74	3.297E-07	1.72	3.50E-07	1.75	1.95E-07
rs11143005 *LOC105376072* (intronic)	TGL	0.420	4.035E-07	0.42	3.218E-07	0.42	2.27E-07	0.41	5.83E-07
chr12:101494770/rs10860880 *IGF1* (downstream)	TGL	1.596	2.077–07	1.60	1.746E-07	1.52	6.21E-07	1.53	5.15E-07
rs900543 *THSD4, NR2E3* (intergenic/downstream to NR2E3)	TGL	1.625	1.27E-07	1.61	1.67E-07	1.58	2.26E-07	1.59	1.849E-07

^@^These markers are as listed in Table 2.

**Table 4:** Markers that achieved suggestive evidence of association with phenotype traits at p-values of <1.0E-05.

Phenotype	Variant	Gene	Model	MAF	Beta$	P-value$	Beta#	P-Value#
WcHtR	rs17117722	*TRA, TRD* (3′UTR)	Additive	0.074	−0.0309	1.07E-06	−0.0303	2.316E-06
HbA1c	rs3767494	*C1orf106* (intronic)	Recessive	0.052	2.971	1.017E-06	2.503	1.557E-05
Weight	rs10005556	*GAPDHP56, LOC105377421* (intergenic)	Additive	0.050	6.63	1.24E-06	6.315	3.308E-06
Weight	rs4559034	*LOC102467224* (intronic)	Additive	0.311	2.901	5.24E-06	2.827	7.99E-06
BMI	rs10005556	*GAPDHP56, LOC105377421* (intergenic)	Additive	0.05	2.21	2.25E-06	2.278	5.03E-06
Waist Circumference	rs10005556	*GAPDHP56, LOC105377421* (intergenic)	Additive	0.05	4.851	1.29E-06	4.729	2.412E-06
Waist Circumference	rs9390649	*UST* (intronic)	Additive	0.36	2.138	1.72E-06	2.112	2.348E-06
HbA1c	rs17639988	*MDGA1, ZFAND3* (intergenic)	Additive	0.083	0.4722	2.21E-06	0.410	1.642E-05
HDL	rs1800775	*CETP* (downstream)	Additive	0.48	−0.0499	1.60E-06	−0.049	1.909E-06
LDL	chr2:43355981	*THADA* (intronic)	Additive	0.38	−0.1383	8.9E-06	−0.124	5.022E-05
TGL	rs9326246	*BUD13* (promoter)	Additive	0.11	0.237	5.19E-06	0.2315	9.853E-06
TGL	rs925530	*TMEM120B* (intronic)	Additive	0.37	0.1574	5.03E-06	0.159	3.668E-06
Total cholesterol	chr2:43355981	*THADA* (intronic)	Additive	0.38	−0.1657	1.37E-06	−0.1494	1.055E-05
Total cholesterol	rs10935794	*RPL32P9, LINC01213* (intergenic)	Additive	0.19	0.2037	3.65E-06	0.2056	2.447E-06
Height	rs3959929	*LOC105376567, LOC105376570* (intergenic)	Recessive	0.065	7.369	6.125E-06	NA@	NA@
Weight	rs1184476	*TEX29* (intergenic)	Recessive	0.055	36.52	1.12E-05	36.74	9.269E-06
HbA1c	chr1:160381119	*LOC105371466*	Recessive	0.053	2.332	3.48E-05	2.548	1.933E-06
HbA1c	rs17716285	*KSR1* (intronic)	Recessive	0.055	3.113	9.87E-06	3.25	1.19E-06
FPG	rs7729384	*LOC101927421* (intronic)	Recessive	0.122	2.169	8.855E-06	1.716	0.000202
FPG	rs3799125	*RGS17* (intronic)	Recessive	0.102	2.599	2.77E-06	2.372	5.906E-06
FPG	rs10247084	*LOC105375159, LOC105375160* (intergenic)	Recessive	0.059	3.865	0.000157	4.373	5.847E-06
FPG	rs883431	*SLC28A3* (upstream)	Recessive	0.184	1.287	3.187E-05	1.394	1.77E-06
FPG	rs747486	*ANKRD11* (intronic)	Recessive	0.076	3.714	2.055E-06	3.187	1.619E-05
FPG	rs4764409	*PIK3C2G* (intronic)	Recessive	0.085	3.314	4.852E-06	3.197	2.95E-06
TGL	rs12722856	*RPS3AP9, GAPDHP75* (intergenic)	Recessive	0.083	1.487	1.313E-06	1.428	3.599E-06
TGL	rs10014125	*LOC105377567* (intronic)	Recessive	0.176	0.6139	1.06E-05	0.6178	9.014E-06
TGL	rs17073574	*LAMA4* (intronic)	Recessive	0.137	0.7335	2.317E-06	0.7258	2.933E-06
TGL	rs11777524	*LY6D, GML* (intergenic)	Recessive	0.052	1.686	2.91E-06	1.692	2.619E-06
TGL	rs11602685	*MICAL2* (intronic)	Recessive	0.099	1.127	5.37E-06	1.125	5.437E-06
TGL	chr11:45779819	*DKFZp779M0652, SLC35C1* (intergenic)	Recessive	0.062	1.91	4.844E-06	1.884	6.319E-06
TGL	rs17569297	*LOC105369738, LOC105369739* (intergenic)	Recessive	0.128	0.773	6.963E-06	0.7732	6.7E-06
TGL	rs7342999	*LOC105372082, LOC105372084* (intergenic)	Recessive	0.129	0.8303	3.953E-06	0.8186	5.261E-06
SBP	rs10497520	*TTN* (coding: K > E)	Recessive	0.155	9.551	5.5E-06	9.339	8.558E-06

^$^Tests for association were adjusted for age, sex, and the first 10 principal components.

^#^Tests for association with anthropometric and lipid traits were adjusted for age, sex, principal components, and Lipid lowering medication status; tests for association with HbA1c and FPG were adjusted for age, sex, principal components, and diabetes medication status; tests for association with SBP and DBP were adjusted for age, sex, principal components, and hypertension medication status.

^@^Tests of association for height were not adjusted for medication.

**Table 5:** Literature evidence for involvement of ‘suggestive’ markers (or gene loci) with p-values of <1.0E-05 in the associated phenotype traits.

Marker/Gene	Phenotype trait	Comments
rs3767494/*C1orf106*	HbA1c	A systematic analysis of genetic loci of cross autoimmune disorders implicated role of *c1orf106* gene in the etiology of Type 1 diabetes^72^.
rs17117722/[*TRA, TRD*]	WcHtR	Both the *TRA* and *TRD* genes are members of the class of T-Cell receptor proteins. T-cells play an important role in the immune adaptation to both obesity and malnutrition^73^. T-cells play an early and critical role in inducing inflammation in obesity and in the accumulation of inflammatory macrophages in obese adipose tissue; both these two processes are known to promote insulin resistance.
rs10005556/*GAPDHP56*	Weight/BMI/Waist Circumference	Genome region of *GAPDHP56* (chr4:131448001-131451000, chr4:131449501-131452500) is reported as associated with total fat mass in genome-wide rare variants analysis of UK10K Cohorts^52^.
rs1184476/*TEX29*	Weight	Variants of *TEX29 (alias C13orf16*) are associated with age at menarche among Chinese women. However, as of now there is no report of their role in Weight gain^53^.
rs9390649/*UST*	Waist circumference	A study using mice suggests an important role for Uronyl 2-sulfotransferase transfers in clearance of triglyceride-rich lipoprotein^74^.
rs17639988/*MDGA1, ZFAND3*	HbA1c	Intergenic SNPs between *MDGA1* and *ZFAND3* are implicated in T2DM in East Asian population^54^.
rs17716285/*KSR1*	HbA1c	Kinase suppressor of Ras 1 (*KSR1*) is required for Ras-mediated ERK pathway activation. This pathway is identified as modifiers of cellular insulin responsiveness. A study conducted on OLETF rat models for T2DM have shown that suppressing the *KSR1/ERK* complex brings down diabetic vascular complications^51^.
rs883431/*SLC28A3*	FPG	A study in rat indicates upregulation of *SLC28A3* in glomerulus in diabetic condition. But till date no human study is reported^49^.
rs4764409/*PIK3C2G*	FPG	Variants from this gene are found to be associated with high HbA1c and low serum insulin levels in Japanese T2D population^55^. *PI3K* orchestrates its action through *AKT2* pathway in the liver, as a main regulator of metabolism^75^.
rs1800775/*CETP*	HDL	rs1800775 is a well-known upstream (~2KB) variant of *CETP* gene shown to be replicated in European population with HDL^76^.
rs9326246/*BUD13*	TGL	Extended CARDIoGRAM study (involving participants of European ancestry) implicated rs9326246 marker as associated with TGL and as one of the important CAD susceptibility loci^76^.
rs925530/*TMEM120B*	TGL	The role of *TMEM120B* in fat metabolism has been established in knockdown cell line studies^50^.
rs17073574/*LAMA4*	TGL	Studies have shown association of *LAMA4* in obesity and diabetic nephropathy^77^,^78^.
rs11777524/*LY6D, GML*	TGL	There is lack of literature reports on the role of *GML* (Glycosylphosphatidylinositol-anchored molecule like) in processes relating to triglyceride. But there is evidence for *GPIHBP1* (Glycosylphosphatidylinositol-anchored high-density lipoprotein-binding protein 1) in causing hypertriglyceridemia. Protein sequence comparison analysis shows 28% identity between these two proteins. Furthermore, it is known that *GPIHBP1* evolved *LY6*-like genes^79^.
rs11602685/*MICAL2*	TGL	Its association with plasma uric acid in obese individuals is evident. However, it requires more literature support to establish its functional role in elevation of triglyceride levels^80^.
rs10497520/*TTN*	SBP	Involvement of the *TTN* gene in hypertension is substantiated by literature evidences. Rain *et al*.^81^ found that phosphorylation of titin in right ventricular tissue was significantly reduced in pulmonary arterial hypertension patients (undergoing heart/lung transplantation) as compared with control subjects. Mutations in this gene are associated with familial hypertrophic cardiomyopathy^82^. Further, Ottenheijm *et al*. demonstrated that alternative splicing of the *TTN* gene was associated with diaphragm dysfunction in patients with mild to moderate chronic obstructive pulmonary disease^83^.

**Table 6:** List of differentially regulated genes as determined by GTEx data analysis on variants associated with metabolic traits.

SNP	Gene mapped	Gene regulated	P-value	Effect Size	Regulation	Tissue
I. Markers associated at close to genome-wide significant p-value of ≤3.41E-08						
Chr15:40531386-rs12440118	*ZNF106* (W > R)	*LRRC57* (downstream of *ZNF106*)	3.7E-09	−0.47	Down	Cells- transformed fibroblast
II. Markers with nominal evidence of association at p-value ≤ 5.45E-07						
rs11143005	*LOC105376072* (intronic)	*LOC105376072* (intronic)	3.3E-08	0.23	Up	Whole Blood
rs11143005	*LOC105376072* (intronic)	*PGM5* (downstream of *LOC105376072*)	4.2E-10	0.50	Up	Whole Blood
III. Markers with suggestive evidence of association at p-value < 1.0E-05						
rs1800775	*CETP* (SNP located downstream of the gene)	*NLRC5* (Upstream of *CETP*)	4.3E-16	−0.35	Down	Cells- transformed fibroblast
rs9326246	*BUD13* (SNP located upstream of the gene)	*RP11-109L13.1* (downstream of *BUD13*)	4.8E-07	−0.80	Down	Skin- Sun exposed
rs17716285	*KSR1* (intronic)	*NOS2* (upstream of *KSR1*)	0.0000051	0.46	Up	Muscle skeletal
rs11777524	*LY6D, GML* (intergenic)	*LOC101928087* (upstream of *LY6D*)	0.0000041	−0.80	Down	Testis
rs10497520	*TTN* (K > E)	*FKBP7* (upstream of *TTN*)	0.000019	−0.19	Down	Cells- transformed fibroblast

**Table 7:** 1000 Genomes Project Phase 3 allele frequencies for markers identified in this study.

SNP	Minor allele frequencies (MAF)@ in					
	AFR (African)	AMR (Ad Mixed American)	EAS (East Asian)	EUR (European)	SAS (South Asian)	Arab (Study population)
rs3767494	*0.02*	0.11	0.13	0.05	0.07	0.053
Chr15:40531386-rs12440118	*0.01*	0.1	*0.01*	0.14	0.07	0.106
rs7144734	0.12	0.2	0.07	0.29	0.17	0.204
rs17501809	*0*	*0.04*	*0*	0.08	*0.04*	0.059
rs11143005	0.42	0.14	*0*	0.19	0.18	0.283
rs900543	0.44	0.12	0.26	*0.04*	0.06	0.087
rs10860880	0.28	*0.04*	*0.01*	*0.02*	*0.04*	0.056
rs17117722	0.34	0.06	*0*	0.06	*0.01*	0.074
rs10005556	0.197	*0.023*	*0*	*0.027*	*0.033*	0.05
rs4559034	0.431	0.157	*0.009*	0.286	0.253	0.31
rs9390649	0.243	0.225	0.271	0.409	0.401	0.36
rs17639988	*0.002*	*0.048*	*0.001*	0.078	*0.034*	0.08
rs1800775	0.607	0.543	0.486	0.488	0.59	0.47
rs9326246	*0.011*	0.133	0.238	0.093	0.193	0.11
rs925530	0.616	0.427	0.451	0.328	0.367	0.37
rs10935794	0.061	0.207	0.326	0.282	0.26	0.19
rs3959929	*0.004*	0.069	*0*	0.11	*0.015*	0.065
rs1184476	0.29	*0.035*	*0*	*0.038*	*0.027*	0.054
chr1:160381119	*0*	*0*	*0*	*0*	*0*	0.053
rs17716285	*0.007*	0.055	*0*	0.093	0.019	0.055
rs7729384	0.066	0.216	0.118	0.242	0.05	0.12
rs3799125	*0.029*	0.086	0.105	0.196	0.098	0.1
rs10247084	0.132	0.085	0.107	0.072	0.077	0.06
rs11785103	*0.003*	*0.027*	*0.001*	*0.044*	*0.039*	0.0642
rs883431	0.402	0.442	0.497	0.172	0.191	0.18
rs4764409	*0.036*	0.411	0.322	0.196	0.194	0.085
rs747486	*0.009*	*0.036*	0.114	0.049	0.134	0.075
rs12722856	*0.005*	*0.029*	*0*	0.067	0.056	0.083
rs10014125	0.365	0.097	0.067	0.132	0.109	0.175
rs17073574	*0.042*	0.082	0.188	0.069	0.116	0.137
rs11777524	0.062	*0.049*	0.325	0.08	0.224	0.052
rs11602685	0.118	0.073	*0.041*	0.095	*0.029*	0.1
chr11:45779819	*0*	*0*	0.168*	0.033*	*0*	0.06
rs17569297	*0.008*	0.072	*0.001*	0.129	0.153	0.13
rs7342999	0.588	0.222	0.202	0.088	0.08	0.13
rs10497520	0.408	0.509	0.217	0.812	0.544	0.155

^@^MAF values less than 5% are shown in italics.

^*^MAF is obtained from Illumina product files (http://support.illumina.com/downloads/cardio-metaboChip_product_support_files.html).
