# Supplementary material for: Genetic risk variants for metabolic traits in Arab populations
Source: Sci Rep. 2017 Jan 20;7:40988. doi: 10.1038/srep40988 (PMC5247683; doi:10.1038/srep40988)
Supplement: Supplementary Information [file srep40988-s1.pdf]

Genetic risk variants for metabolic traits in Arab populations.

Prashantha Hebbar[@], Naser Elkum[@], Fadi Alkayal, Sumi Elsa John, Thangavel Alphonse Thanaraj[*], Osama Alsmadi[*]

Dasman Diabetes Institute, P.O. Box 1180, Dasman 15462, Kuwait

[@],These two authors contributed equally to the study and hence may be considered as joint first authors.

[*], These two authors contributed equally to this study. Correspondence may be addressed to either of the two authors:

Osama Alsmadi, PhD

Dasman Diabetes Institute

P.O. Box 1180, Dasman 15462, Kuwait.

Tel (work): +965 2224 2999 Ext. 4343

Fax: +965 2249 2406

E-mail: **oalsmadi@gmail.com**

Thangavel Alphonse Thanaraj, M.Sc., PhD.,

Dasman Diabetes Institute,

P.O. Box 1180, Dasman 15462, Kuwait.

Phone: +965 2224 2999 Ext. 3329

Fax: +965 2249 2436

Email: **alphonse.thangavel@dasmaninsitute.org**

SUPPLEMENTARY INFORMATION

List of Supplementary Figures

Supplementary Figure S1. Scatter plot representing the first two principal components of the sample set.

Supplementary Figure S2. Quantile-Quantile plots of the expected and observed –log P values for SNP associations with the phenotype traits of **(A)** HbA1c-recessive, **(B)** FPG-recessive, and **(C)** TGL-recessive.

Supplementary Figure S3. Regional association plots, showing individual SNPs in the gene regions and their association with the phenotype traits. The association levels are given along the left Y-axis. SNPs from the region are colored based on their r^2 with the top hit SNP which has the smallest P value in the region. **(A)** *ZNF106* with HbA1c; **(B)** [*OTX2-AS1*] with FPG; **(C)** *PLGRKT* with TGL; **(D)** *LOC105376072* with TGL; **(E)** [*THSD4, NR2E3*] with TGL; and **(F)** *IGF1* with TGL. In the case of the regional plot for *IGF1*, there are two dots at top or borderline p-values; the variant Chr12:101499141 is in LD (r^2= 0.97) with chr12:101494770.

List of Supplementary Tables

Supplementary Table S1. Genomic control inflation factors corresponding to the QQ-plots.

Supplementary Table S2. Results of examining the identified markers for associations with respective traits in EBI GWAS Catalog. The table was created by filtering the GWAS Catalog for the phenotype trait to which the marker was seen associated in our study. Supplementary Dataset 1 lists the results from GWAS Catalog for all the identified markers against all the phenotype traits reported in the study.

Supplementary Table S3. Results of examining the identified markers for associations with related metabolic traits in EBI GWAS Catalog.

Supplementary Table S4. Performance of most replicated exemplary gene loci (*PPARg, KCNJ11, TCF7L2, SLC30A, ABCC8, HHEX, CDKN2A, IGF2BP2, CDKAL1,* and *FTO*) relating to obesity and diabetes in our study population. Listed are only those markers for which a p-value of ≤0.05 was observed in our MetaboChip data. None of the markers from the *ABCC8, HHEX, CDKN2A, IGF2BP2,* and *FTO* genes surfaced with a p-value of ≤0.05.

Supplementary Figure S1. Scatter plot representing the first two principal components of the sample set.

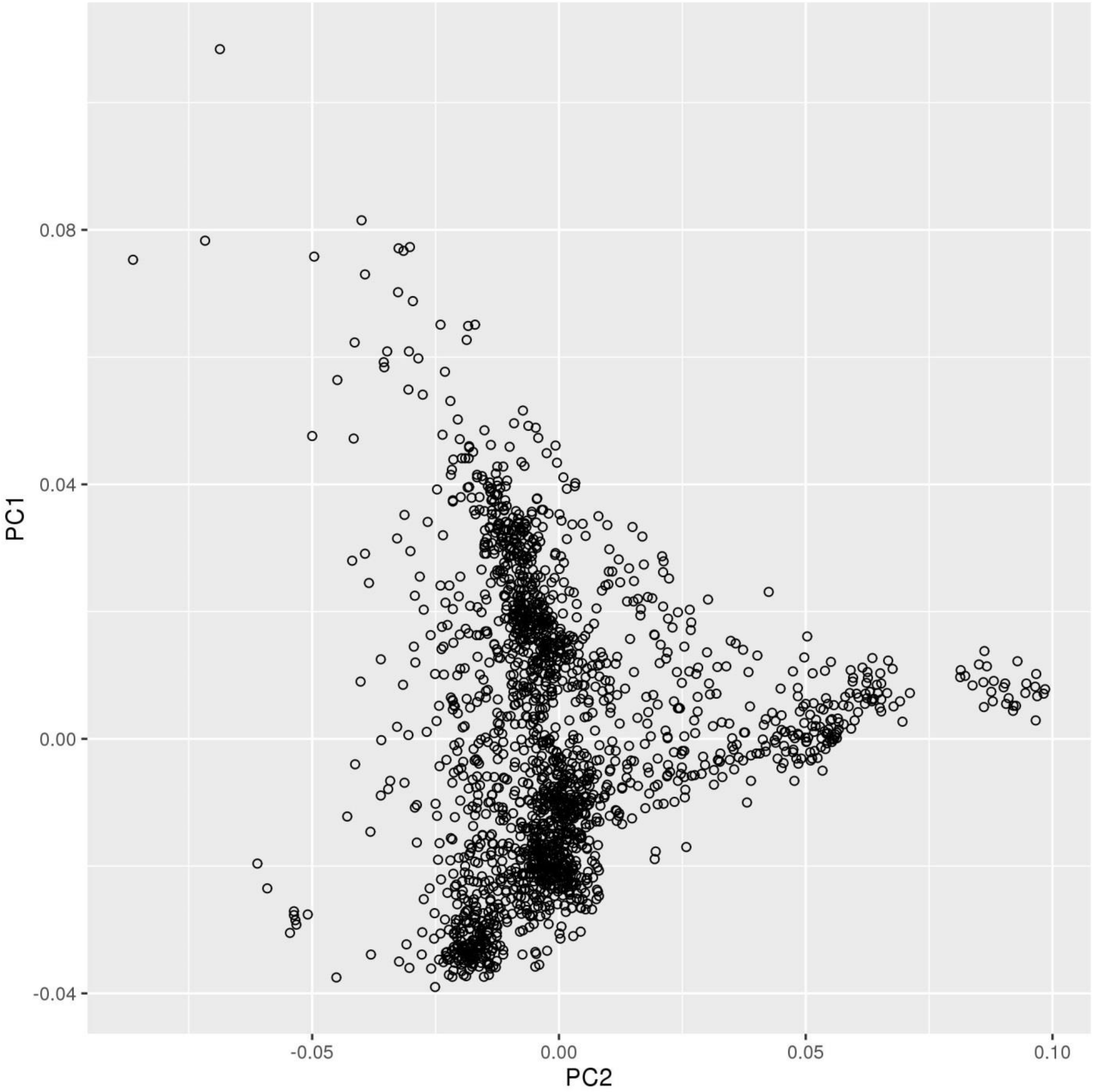

Supplementary Figure S2. Quantile-Quantile plots of the expected and observed –log P values for SNP associations with the phenotype traits of **(A)** HbA1c-recessive, **(B)** FPG-recessive, and **(C)** TGL-recessive.

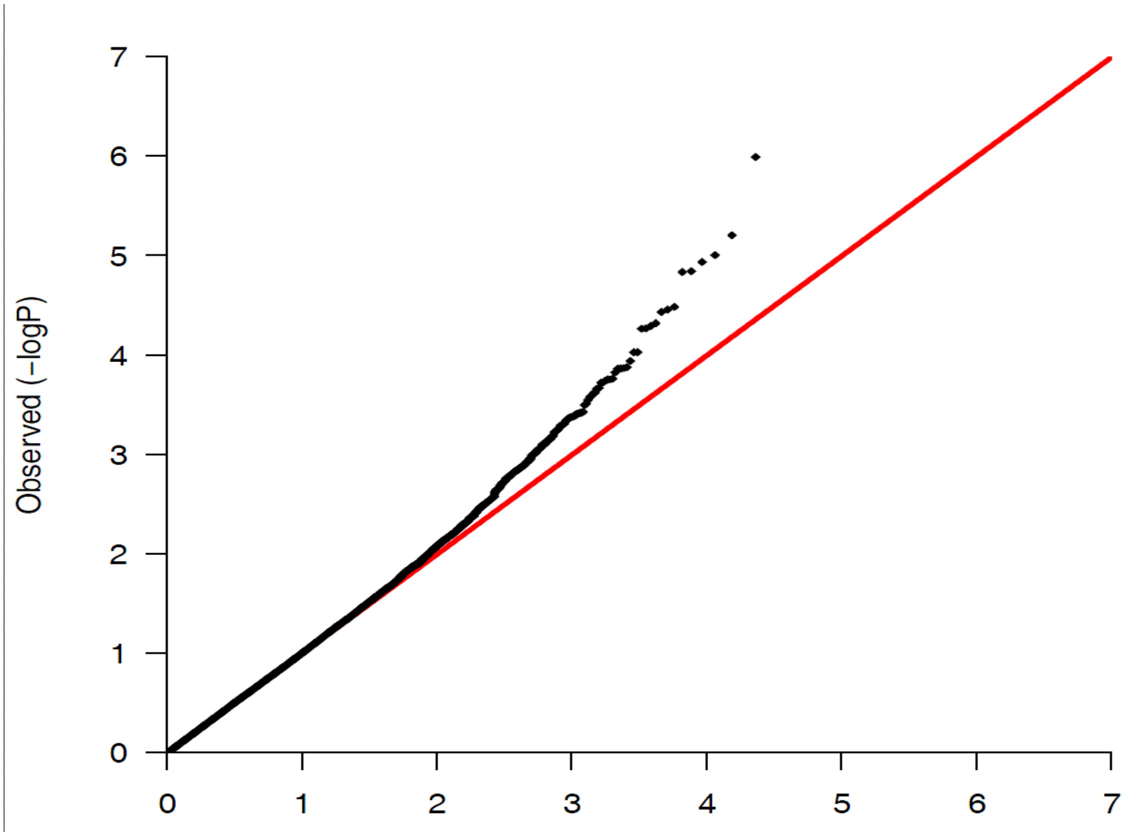

Supplementary Figure S2.A. The -log10 of p-values observed for the association of SNPs with HbA1c in merged data analysis under recessive model adjusted for age, sex, first 10 PC are plotted against the theoretical -log10 p-values expected under the null hypothesis (red line).

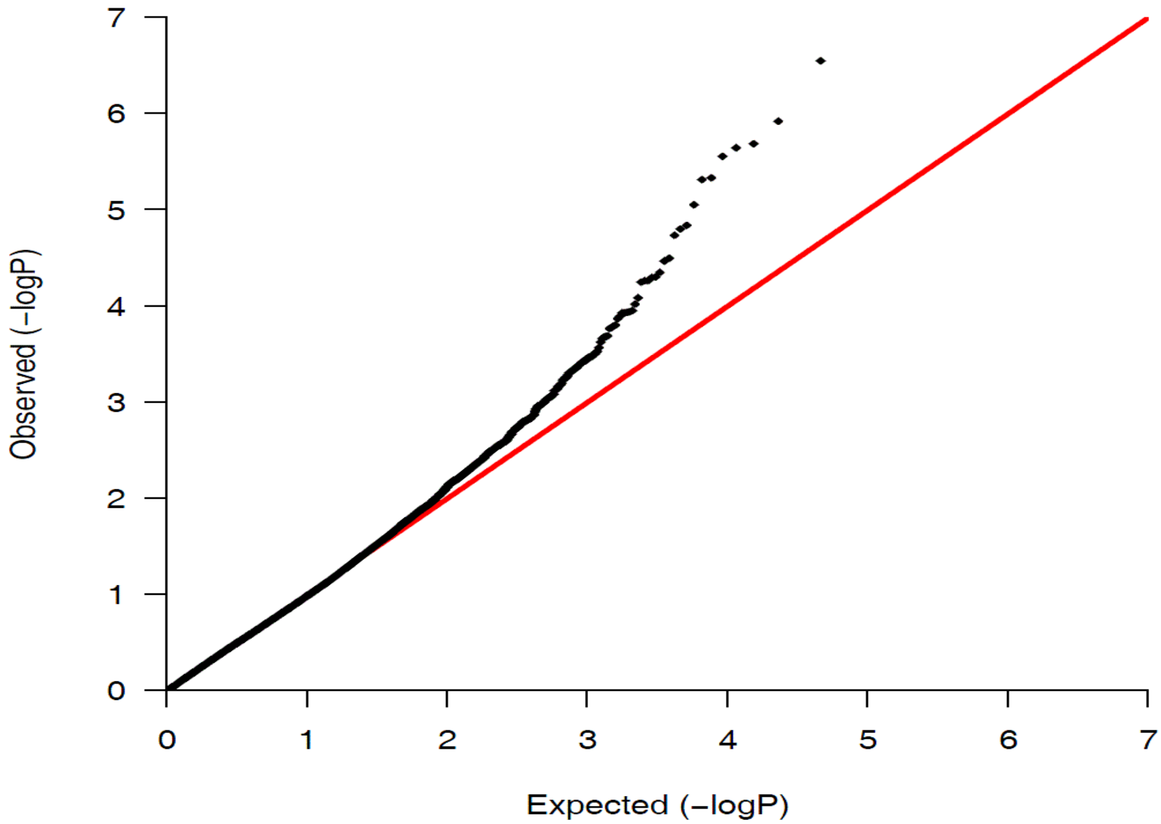

Supplementary Figure S2.B. The -log10 of p-values observed for the association of SNPs with FPG in merged data analysis under additive model adjusted for age, sex, first 10 PC are plotted against the theoretical -log10 p-values expected under the null hypothesis (red line).

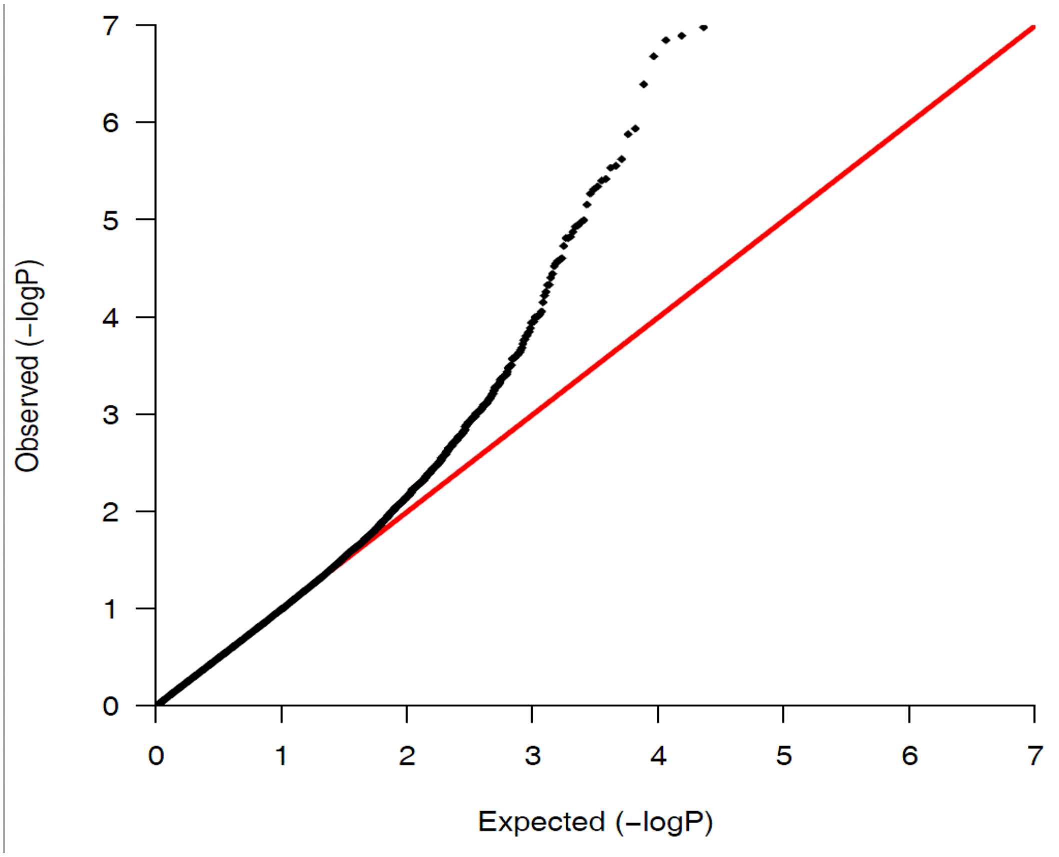

Supplementary Figure S2.C. The -log10 of p-values observed for the association of SNPs with TGL in merged data analysis under recessive model adjusted for age, sex, first 10 PC are plotted against the theoretical -log10 p-values expected under the null hypothesis (red line).

Supplementary Figure S3. Regional association plots, showing individual SNPs in the gene regions and their association with the phenotype traits. The association levels are given along the left Y-axis. SNPs from the region are colored based on their r^2 with the top hit SNP which has the smallest *P* value in the region. **(A)** *ZNF106* with HbA1c; **(B)** [*OTX2-AS1*] with FPG; **(C)** *PLGRKT* with TGL; **(D)** *LOC105376072* with TGL; **(E)** [*THSD4, NR2E3*] with TGL; and **(F)** *IGF1* with TGL. In the case of the regional plot for *IGF1*, there are two dots at top or borderline p-values; the variant Chr12:101499141 is in LD (r^2= 0.97) with chr12:101494770.

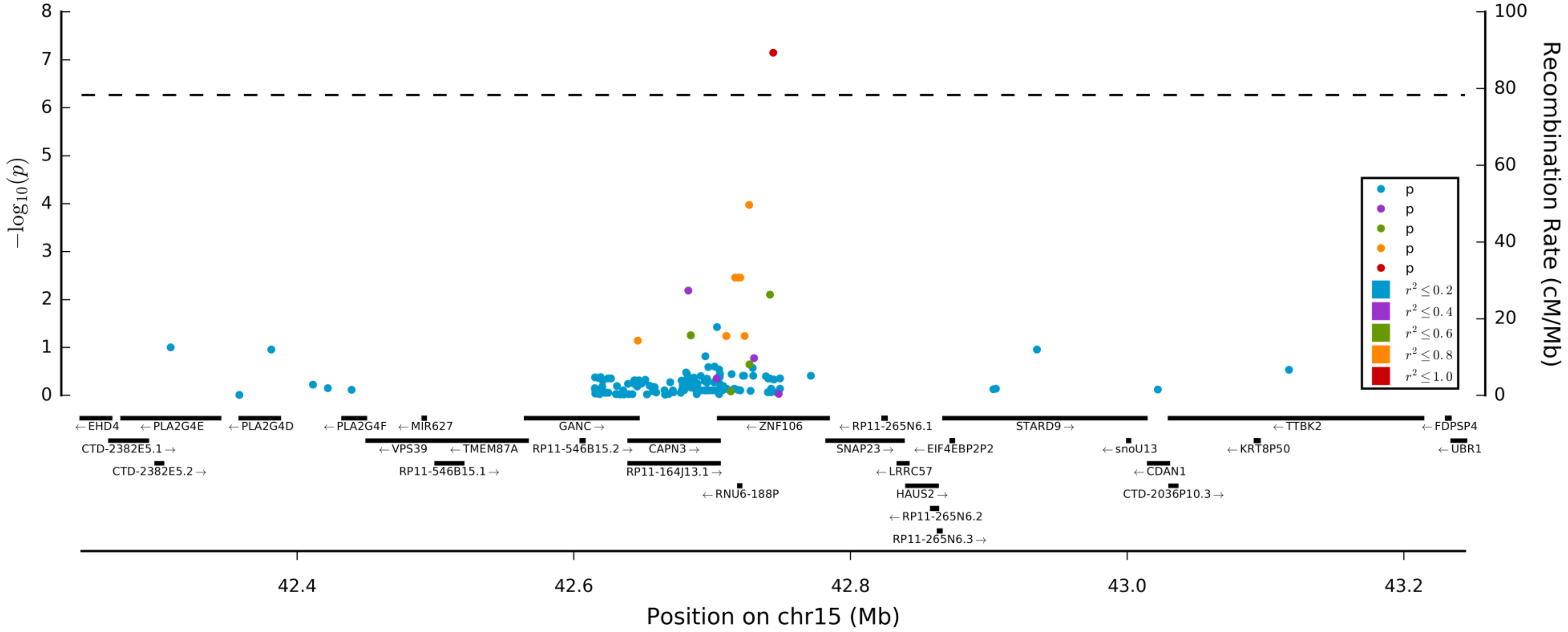

Supplementary Figure S3.A. Regional association plot for SNP chr15:40531386/rs12440118 (*ZNF106*) with HbA1c associated region. Linkage disequilibrium (LD) is indicated by color scale in relationship to marker. The color scheme is red for strong LD (r^2 >0.8) and orange, green, purple, blue colors for lower LD.

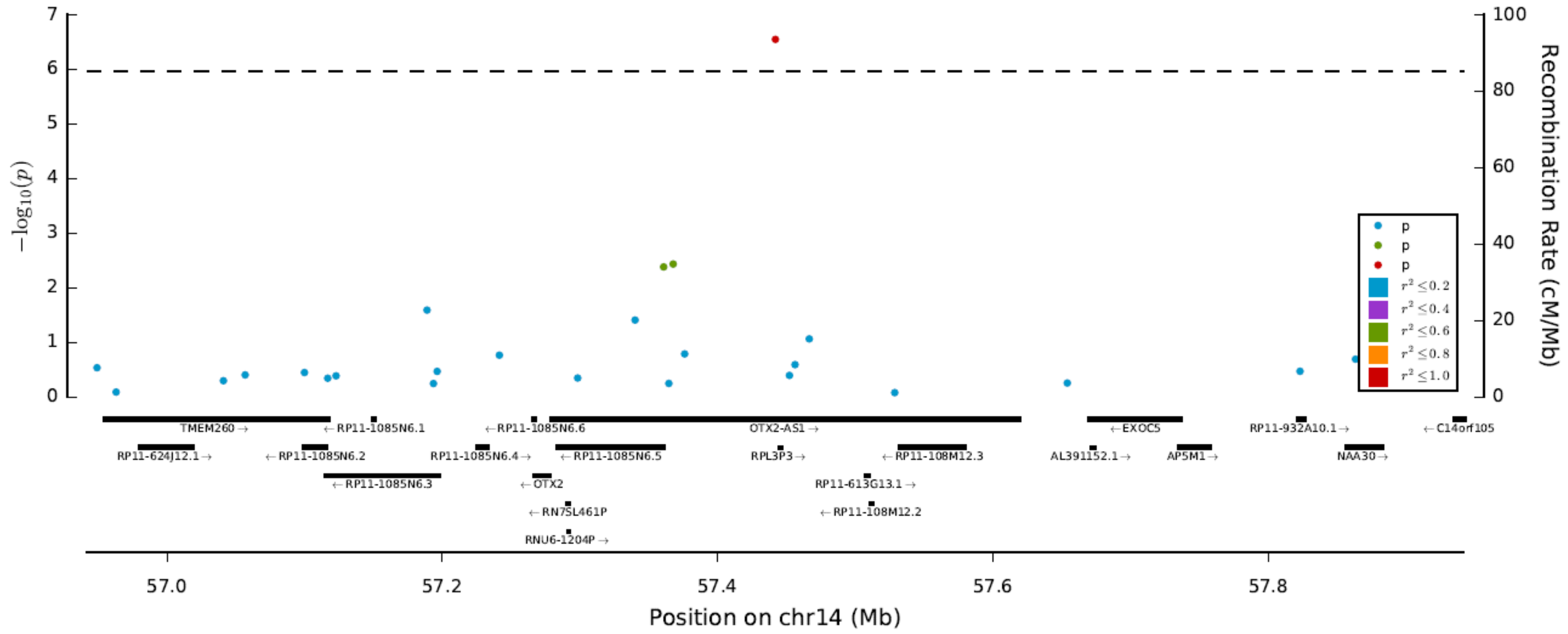

Supplementary Figure S3.B. Regional association plot for SNP rs7144734 (*OTX2-AS1*) with FPG associated region. Linkage disequilibrium (LD) is indicated by color scale in relationship to marker. The color scheme is red for strong LD (r^2 >0.8) and orange, green, purple, blue colors for lower LD.

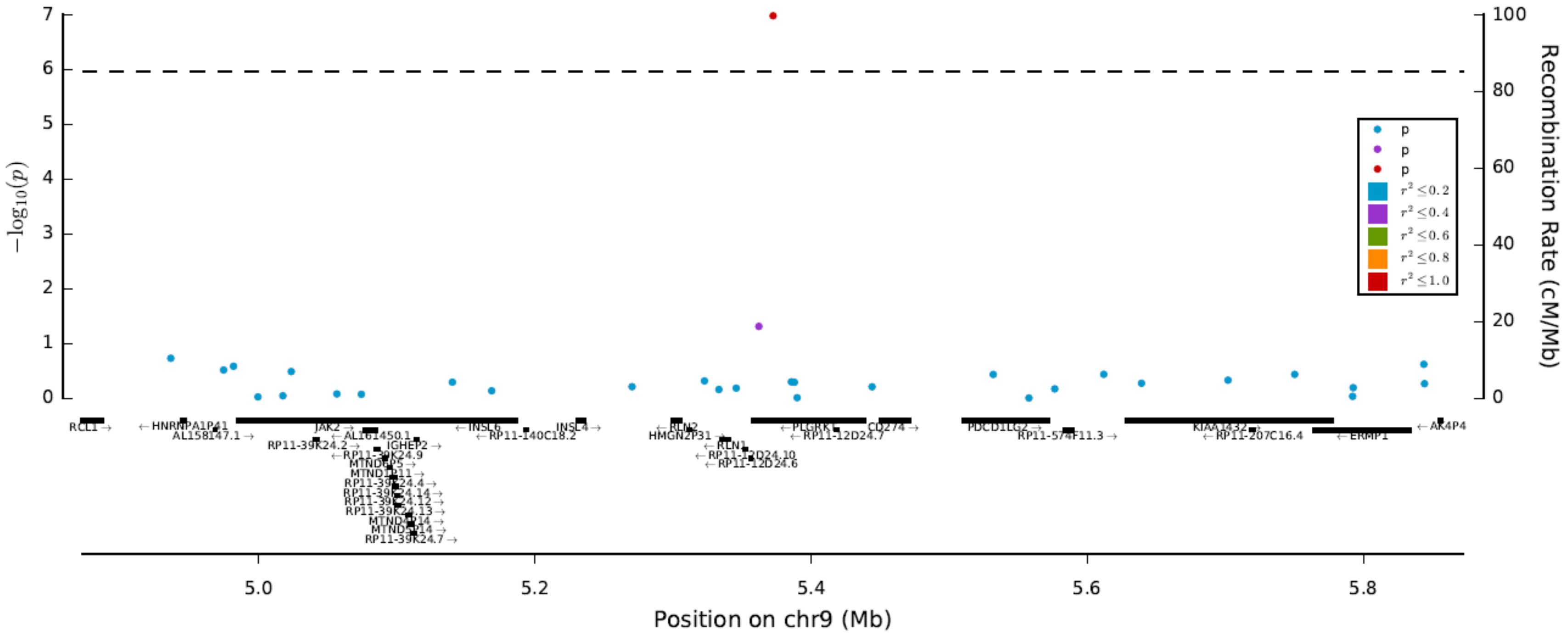

Supplementary Figure S3.C. Regional association plot for SNP rs17501809 (*PLGRKT*) with TGL associated region. Linkage disequilibrium (LD) is indicated by color scale in relationship to marker. The color scheme is red for strong LD (r^2 >0.8) and orange, green, purple, blue colors for lower LD.

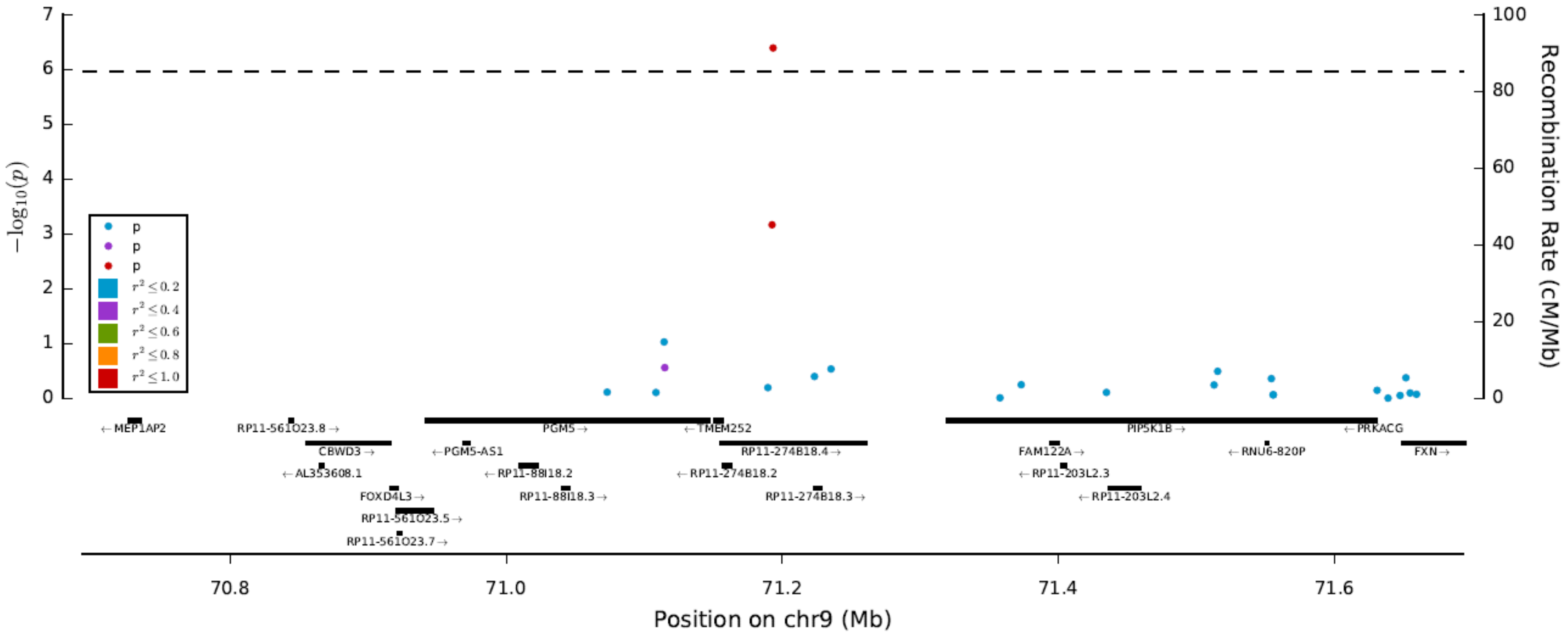

Supplementary Figure S3.D. Regional association plot for SNP rs11143005 (*LOC105376072*) with TGL associated region. Linkage disequilibrium (LD) is indicated by color scale in relationship to marker. The color scheme is red for strong LD (r^2 >0.8) and orange, green, purple, blue colors for lower LD.

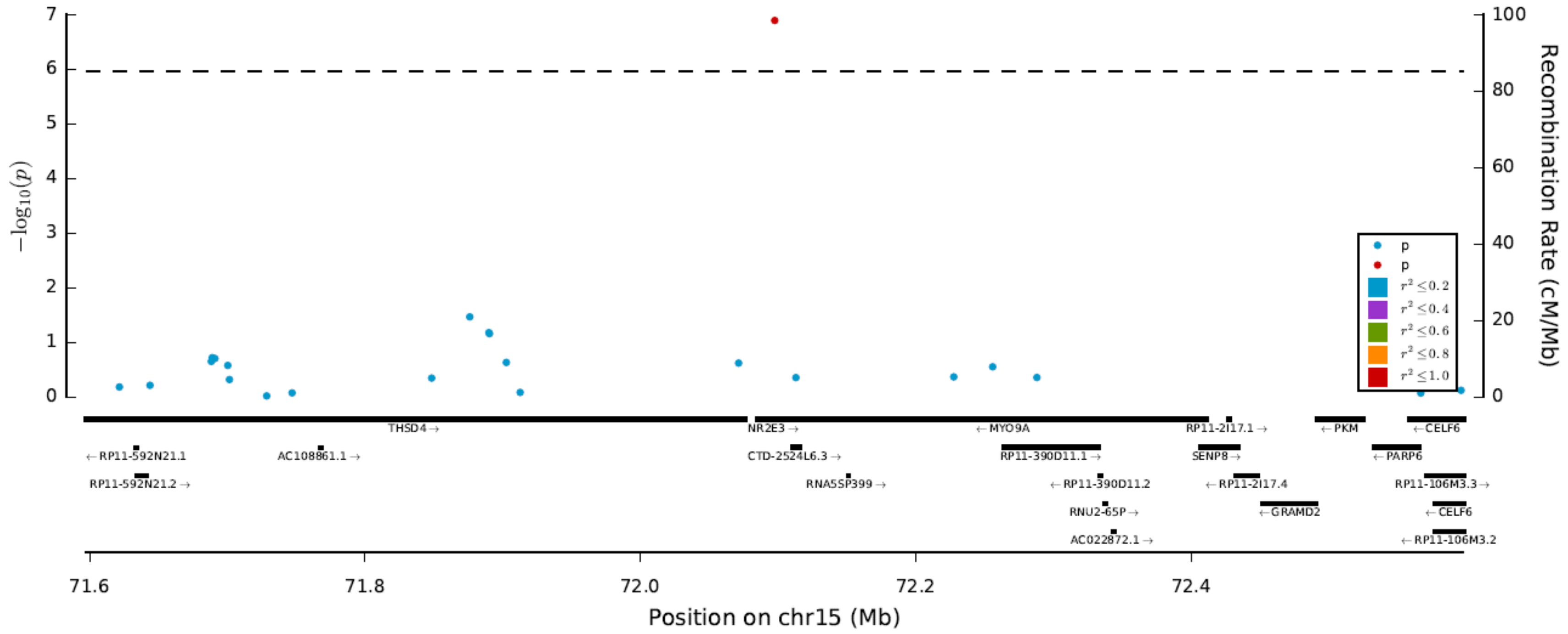

Supplementary Figure S3.E. Regional association plot for rs900543 (*THSD4-NR2E3*) with TGL associated region. Linkage disequilibrium (LD) is indicated by color scale in relationship to marker. The color scheme is red for strong LD (r^2 >0.8) and orange, green, purple, blue colors for lower LD.

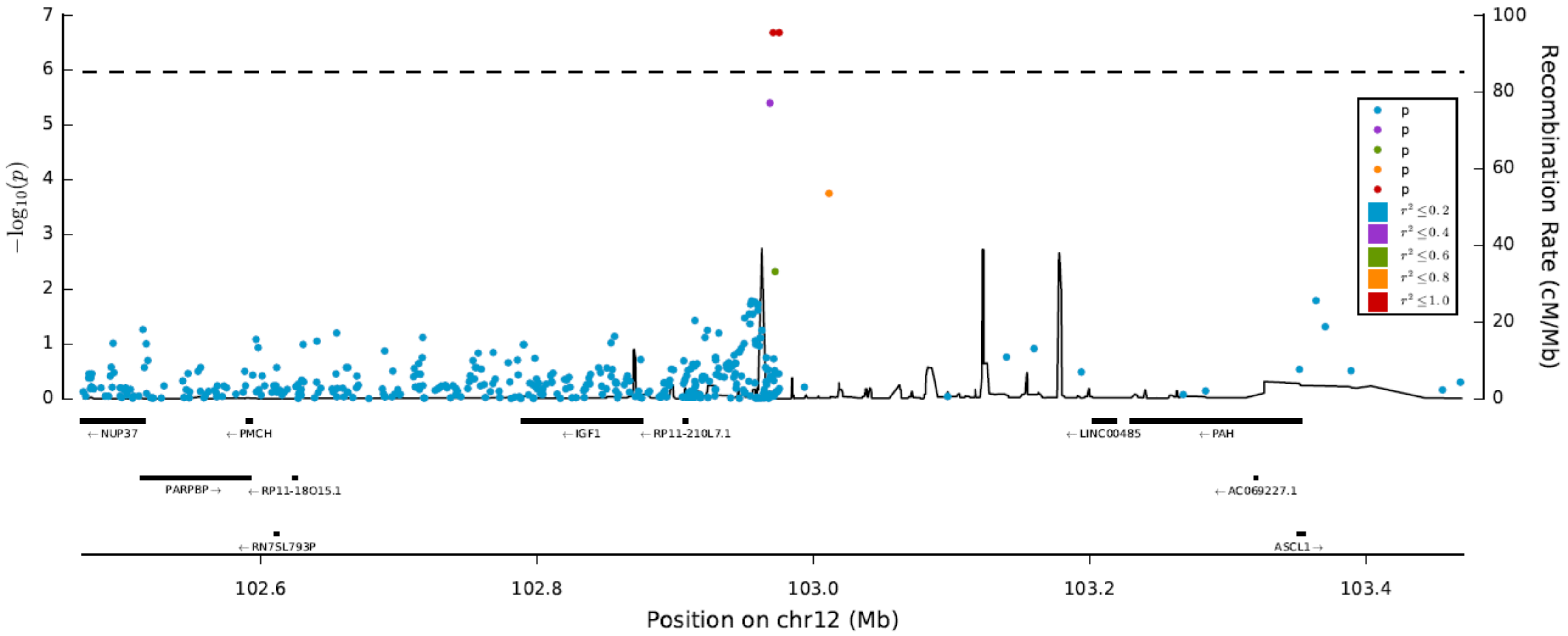

Supplementary Figure S3.F. Regional association plot for chr12:101494770 (*IGF1*) with TGL associated region. The variant Chr12:101499141 is in LD (R-Sq = 97%) with chr12:101494770. Linkage disequilibrium (LD) is indicated by color scale in relationship to marker. The color scheme is red for strong LD (r^2 >0.8) and orange, green, purple, blue colors for lower LD.

Supplementary Table S1. Genomic control inflation factors corresponding to the tested 13 metabolic traits under additive and recessive mode of inheritance.

Metabolic trait	λ for the association tests with the *regular adjustment* (for age, sex, and principal components)	λ for the association tests with the *regular adjustment* and further adjustment for medication[#]
**RECESSIVE MODEL**		
Weight	1.00	1.00
WcHtR	0.94	0.94
WC	1.00	1.00
TGL	1.00	1.00
TC	0.99	0.98
HDL	0.99	1.00
LDL	1.00	1.00
BMI	1.00	1.00
Height	1.03	NA
HbA1c	1.02	1.00
FPG	1.02	1.00
SBP	1.00	0.99
DBP	1.00	1.00
**ADDITIVE MODEL**		
Weight	1.01	1.01
WcHtR	0.98	0.98
WC	1.00	1.00
TGL	1.00	1.01
TC	0.99	0.98
HDL	1.00	0.99
LDL	0.99	1.00
BMI	1.00	1.01

Height	1.04	-
HbA1c	1.02	1.02
FPG	1.00	1.00
SBP	0.99	0.99
DBP	1.01	1.01

#, Tests for association with anthropometric and lipid traits were adjusted for age, sex, principal components, and Lipid lowering medication status; tests for association with HbA1c and FGL were adjusted for age, sex, principal components, and diabetes lowering medication status; tests for association with SBP and DBP were adjusted for age, sex, principal components, and hypertension lowering medication status.

Supplementary Table S2. Results of examining the identified markers for associations with respective traits in EBI GWAS Catalog. The table was created by filtering the GWAS Catalog for the phenotype trait to which the marker was seen associated in our study. Supplementary Dataset 1 lists the results from GWAS Catalog for all the identified markers against all the phenotype traits reported in the study.

Marker	Gene	Trait	Model	Population	MAF	Beta	p-value	Reference$
**A. Comparisons for the markers that appeared at close to genome-wide significant p-values in our data**								
chr15:40531386 / rs12440118	*ZNF106* (W>R)	HbA1c	REC	This study	0.1059	2.006	7.07E-08	
		FPG		European	0.279	0.0039	0.3357	Soranzo N *et al.*[1]
**B. Comparison of markers that appeared at nominally significant p-values in our data**								
rs7144734	*OTX2-AS1* (upstream)	FPG	REC	This study	0.2036	1.465	2.825E-07	
		HbA1C		European	0.279	0.0119	0.001659	*Soranzo N et al.*[1]
		FPG		European	0.279	0.0039	0.3357	*Dupuis J. et al.*[2]
		FPG		European	0.279	0.0068	0.7653	*Scott RA et al.*[3]
rs17501809	*PLGRKT* (intronic)	TGL	REC	This study	0.0593	1.807	1.043E-07	
		TGL		European	0.0739	8.00E-04	0.8932	*Willer CJ et al.*[4]
		TGL		Mixed	NA	0.0108	0.4517	*Teslovich TM et al.*[5]
rs11143005	*LOC105376072* (intronic)	TGL	REC	This study	0.2829	0.4196	4.035E-07	
		TGL		European	0.1847	-0.0113	0.04612	*Willer CJ et al.*[4],
		TGL		Mixed	NA	-0.0134	0.07292	*Teslovich TM et al.*[5]
chr12:101494770/ rs10860880	*IGF1* (downstream)	TGL	REC	This study	0.05575	1.596	2.077-07	
		TGL		European	0.0238	-0.0257	0.3573	*Willer CJ et al.*[4]

rs900543	*THSD4, NR2E3* (intergenic/downstream to *NR2E3*)	TGL	REC	This study	0.08696	1.625	1.27E-07	
		TGL		European	0.0475	-0.0028	0.4488	*Willer CJ et al.*[4]
		TGL		Mixed	NA	-0.0111	0.2208	*Teslovich TM et al*[5]*.*
**C. Markers that appeared with suggestive evidence of association in our data but are seen at genome-wide significant p-values in EBI GWAS Catalog**								
rs1800775	*CETP* (downstream)	HDL	ADD	This study	0.48	-0.0499	1.60E-06	
		HDL		European	0.48	0.2022	3.33E-644	*Willer CJ et al.*[4]
		HDL		Mixed	NA	0.196	2.06E-306	*Teslovich TM et al.*[5]
rs9326246	*BUD13* (promoter)	TGL	ADD	This study	0.11	0.237	5.19E-06	
		TGL		European	0.0937	0.2185	1.27E-229	*Willer CJ et al.*[4]
		TGL		European		NA	6.70E-32	*Kathiresan S et al.*[6]
		TGL		Mixed		0.2185	4.79E-124	*Teslovich TM et al.*[5]
		TGL		Mixed		NA	4.70E-124	*Deloukas P et al.*[7]

$^{\$}$, Full citations to the references are as listed below:

1 Soranzo, N. *et al.* Common variants at 10 genomic loci influence hemoglobin A(1)(C) levels via glycemic and nonglycemic pathways. *Diabetes* **59**, 3229-3239, doi:10.2337/db10-0502 (2010).

2 Dupuis, J. *et al.* New genetic loci implicated in fasting glucose homeostasis and their impact on type 2 diabetes risk. *Nat Genet* **42**, 105-116, doi:10.1038/ng.520 (2010).

3 Scott, R. A. *et al.* Large-scale association analyses identify new loci influencing glycemic traits and provide insight into the underlying biological pathways. *Nat Genet* **44**, 991-1005, doi:10.1038/ng.2385 (2012).

4 Willer, C. J. *et al.* Discovery and refinement of loci associated with lipid levels. *Nat Genet* **45**, 1274-1283, doi:10.1038/ng.2797 (2013).

5 Teslovich, T. M. *et al.* Biological, clinical and population relevance of 95 loci for blood lipids. *Nature* **466**, 707-713, doi:10.1038/nature09270 (2010).
6 Kathiresan, S. *et al.* Common variants at 30 loci contribute to polygenic dyslipidemia. *Nat Genet* **41**, 56-65, doi:10.1038/ng.291 (2009).
7 Deloukas, P. *et al.* Large-scale association analysis identifies new risk loci for coronary artery disease. *Nat Genet* **45**, 25-33, doi:10.1038/ng.2480 (2013).

Supplementary Table S3. Results of examining the identified markers for associations with related metabolic traits in EBI GWAS Catalog.

SNP	Associated trait in our study	P-value (and beta value) from our study	Associated trait in GWAS Catalog	P-value (and beta value) from GWAS Catalog	Population group	Source	Reference
rs900543/[*THSD4,NR2E3*]	TGL	2.26E-07 (1.625)	Fasting insulin	9.40E-05 (0.036)	European	MAGIC and NHLBI GRASP	*Dupuis, J. et al.*[1]
rs11143005/*LOC105376072*	TGL	3.218E-07 (0.420)	2 hour fasting glucose	4.47E-05 (0.11)	European	MAGIC and NHLBI GRASP	*Saxena, R. et al.*[2]
rs17569297/[*LOC105369738, LOC105369739*]	TGL	6.963E-06 (0.773)	HDL	1.51E-06 (NA)	European	NHLBI GRASP	*Heid, I. M. et al.*[3]
rs10935794/[*RPL32P9, LINC01213*]	Total cholesterol	3.65E-06 (0.2037)	Serum ratio of arabinosefructose	9.80E-05 (NA)	European	NHLBI GRASP	*Suhre, K. et al.*[4]

1. Dupuis, J. *et al.* New genetic loci implicated in fasting glucose homeostasis and their impact on type 2 diabetes risk. *Nat Genet* **42**, 105-116, doi:10.1038/ng.520 (2010).
2. Saxena, R. *et al.* Genetic variation in GIPR influences the glucose and insulin responses to an oral glucose challenge. *Nat Genet* **42**, 142-148, doi:10.1038/ng.521 (2010)
3. Heid, I. M. *et al.* Genome-wide association analysis of high-density lipoprotein cholesterol in the population-based KORA study sheds new light on intergenic regions. *Circ Cardiovasc Genet* **1**, 10-20, doi:10.1161/CIRCGENETICS.108.776708 (2008).
4. Suhre, K. *et al.* Human metabolic individuality in biomedical and pharmaceutical research. *Nature* **477**, 54-60, doi:10.1038/nature10354 (2011)

Supplementary Table S4. Performance of most replicated exemplary gene loci (*PPARg, KCNJ11, TCF7L2, SLC30A, ABCC8, HHEX, CDKN2A, IGF2BP2, CDKAL1,* and *FTO*) relating to obesity and diabetes in our study population. Listed are only those markers for which a p-value of ≤0.05 was observed in our MetaboChip data. None of the markers from the *ABCC8, HHEX, CDKN2A, IGF2BP2,* and *FTO* genes surfaced with a p-value of ≤0.05.

Chr	SNP	Gene	Phenotype	Model	Allele Associated	MAF	Beta	P-Value
6	chr6:20551661	*CDKAL1*	FPG	ADD	G	0.06215	0.5341	0.004006
6	chr6:20637632	*CDKAL1*	FPG	ADD	A	0.09412	-0.389	0.01072
6	chr6:20721906	*CDKAL1*	FPG	ADD	G	0.324	0.191	0.04264
6	chr6:21174762	*CDKAL1*	FPG	ADD	G	0.354	-0.1877	0.04084
6	chr6:21189629	*CDKAL1*	FPG	ADD	G	0.1641	-0.3122	0.00859
6	chr6:21203890	*CDKAL1*	FPG	ADD	G	0.08474	0.35	0.02761
6	chr6:21208023	*CDKAL1*	FPG	ADD	G	0.2153	-0.2288	0.03427
6	chr6:20572488	*CDKAL1*	DBP	ADD	G	0.3821	0.7531	0.03343
6	chr6:20590390	*CDKAL1*	DBP	ADD	G	0.389	0.7514	0.03182
6	chr6:20634996	*CDKAL1*	DBP	ADD	A	0.3675	0.7694	0.03209
6	rs7738382	*CDKAL1*	DBP	ADD	C	0.3514	0.8257	0.0214
6	chr6:20861562	*CDKAL1*	DBP	ADD	A	0.09668	1.479	0.01002
6	chr6:20915183	*CDKAL1*	DBP	ADD	G	0.169	1.145	0.0121
6	chr6:20916721	*CDKAL1*	DBP	ADD	A	0.4739	-0.6974	0.0392
6	chr6:20976273	*CDKAL1*	DBP	ADD	G	0.168	1.133	0.01421
6	chr6:21046566	*CDKAL1*	DBP	ADD	G	0.1092	1.526	0.005071
6	chr6:21104054	*CDKAL1*	DBP	ADD	A	0.08824	1.759	0.002897
6	chr6:20595229	*CDKAL1*	BMI	ADD	A	0.2409	0.5688	0.01815
6	chr6:20636813	*CDKAL1*	BMI	ADD	A	0.1345	0.6599	0.0299
6	chr6:20824232	*CDKAL1*	BMI	ADD	A	0.3358	-0.5413	0.01356
6	chr6:20932202	*CDKAL1*	BMI	ADD	G	0.1013	-0.7268	0.03278
6	rs9350294	*CDKAL1*	BMI	ADD	A	0.4269	0.4517	0.03009
6	chr6:21024416	*CDKAL1*	BMI	ADD	G	0.1031	-0.7342	0.03383

6	chr6:21063311	*CDKAL1*	BMI	ADD	G	0.2954	0.5595	0.01202
6	chr6:21116822	*CDKAL1*	BMI	ADD	G	0.1573	-0.8327	0.00312
6	chr6:21116972	*CDKAL1*	BMI	ADD	A	0.4527	-0.4741	0.02109
6	chr6:21170579	*CDKAL1*	BMI	ADD	C	0.0914	-1.092	0.002474
6	chr6:20551661	*CDKAL1*	HbA1c	ADD	G	0.06215	0.2653	0.02825
6	chr6:21090584	*CDKAL1*	HbA1c	ADD	A	0.07011	0.2123	0.04776
6	chr6:21115720	*CDKAL1*	HbA1c	ADD	A	0.4442	-0.1551	0.006613
6	chr6:21145188	*CDKAL1*	HbA1c	ADD	A	0.009463	-0.6419	0.02844
6	chr6:21174762	*CDKAL1*	HbA1c	ADD	G	0.354	-0.1797	0.002768
6	chr6:21189629	*CDKAL1*	HbA1c	ADD	G	0.1641	-0.1773	0.02308
6	chr6:20608501	*CDKAL1*	HDL	ADD	T	0.09795	0.03835	0.03541
6	chr6:20932202	*CDKAL1*	HDL	ADD	G	0.1013	-0.03682	0.03453
6	chr6:21024416	*CDKAL1*	HDL	ADD	G	0.1031	-0.0436	0.01385
6	chr6:21170579	*CDKAL1*	HDL	ADD	C	0.0914	0.04603	0.01298
6	chr6:20839730	*CDKAL1*	Height	ADD	G	0.08414	0.8202	0.02451
6	chr6:20626004	*CDKAL1*	LDL	ADD	T	0.06189	-0.1351	0.04264
6	chr6:20637632	*CDKAL1*	LDL	ADD	A	0.09412	0.1252	0.02128
6	chr6:20653447	*CDKAL1*	LDL	ADD	G	0.4657	-0.0636	0.04458
6	chr6:20710359	*CDKAL1*	LDL	ADD	G	0.409	0.08316	0.009617
6	chr6:20742594	*CDKAL1*	LDL	ADD	A	0.07446	0.1368	0.025
6	rs6928012	*CDKAL1*	LDL	ADD	G	0.4373	-0.06932	0.03167
6	chr6:21056750	*CDKAL1*	LDL	ADD	C	0.07621	0.1595	0.007534
6	chr6:21113741	*CDKAL1*	LDL	ADD	C	0.08977	0.1152	0.03489
6	chr6:20634996	*CDKAL1*	SBP	ADD	A	0.3675	1.018	0.0486
6	chr6:20688545	*CDKAL1*	SBP	ADD	G	0.1389	1.653	0.02138
6	chr6:20820440	*CDKAL1*	SBP	ADD	G	0.2005	-1.445	0.02016
6	chr6:20861562	*CDKAL1*	SBP	ADD	A	0.09668	1.747	0.03434
6	chr6:20974836	*CDKAL1*	SBP	ADD	G	0.4095	1.181	0.01748
6	chr6:20998360	*CDKAL1*	SBP	ADD	G	0.01969	4.308	0.01313
6	chr6:21006119	*CDKAL1*	SBP	ADD	G	0.4136	1.046	0.03696

6	chr6:20637632	*CDKAL1*	TC	ADD	A	0.09412	0.1368	0.02292
6	chr6:20653447	*CDKAL1*	TC	ADD	G	0.4657	-0.07517	0.03144
6	chr6:20710359	*CDKAL1*	TC	ADD	G	0.409	0.09965	0.004779
6	chr6:20737560	*CDKAL1*	TC	ADD	G	0.1606	-0.09607	0.04316
6	rs6928012	*CDKAL1*	TC	ADD	G	0.4373	-0.08995	0.01115
6	chr6:20864955	*CDKAL1*	TC	ADD	C	0.0954	-0.1414	0.01692
6	chr6:21056750	*CDKAL1*	TC	ADD	C	0.07621	0.1505	0.0225
6	chr6:21189629	*CDKAL1*	TC	ADD	G	0.1641	-0.1159	0.01363
6	chr6:20682542	*CDKAL1*	TGL	ADD	G	0.09488	0.1104	0.04372
6	chr6:21052580	*CDKAL1*	TGL	ADD	T	0.4629	-0.06782	0.03649
6	chr6:21099063	*CDKAL1*	TGL	ADD	A	0.4655	-0.06672	0.03948
6	chr6:21116972	*CDKAL1*	TGL	ADD	A	0.4527	-0.08319	0.01058
6	chr6:21170579	*CDKAL1*	TGL	ADD	C	0.0914	-0.1406	0.01411
6	chr6:21174762	*CDKAL1*	TGL	ADD	G	0.354	-0.07467	0.02944
6	chr6:21189629	*CDKAL1*	TGL	ADD	G	0.1641	-0.09132	0.03947
6	chr6:21208023	*CDKAL1*	TGL	ADD	G	0.2153	-0.1026	0.01086
6	chr6:20824232	*CDKAL1*	WC	ADD	A	0.3358	-1.165	0.01297
6	chr6:21063311	*CDKAL1*	WC	ADD	G	0.2954	1.106	0.02007
6	chr6:21116822	*CDKAL1*	WC	ADD	G	0.1573	-1.558	0.01
6	chr6:21170579	*CDKAL1*	WC	ADD	C	0.0914	-2.172	0.005095
6	chr6:20572488	*CDKAL1*	WcHtR	ADD	G	0.3821	-0.00738	0.03883
6	rs7738382	*CDKAL1*	WcHtR	ADD	C	0.3514	-0.00746	0.03919
6	chr6:20872451	*CDKAL1*	WcHtR	ADD	A	0.1399	-0.01129	0.02193
6	chr6:20932202	*CDKAL1*	WcHtR	ADD	G	0.1013	-0.01115	0.04887
6	chr6:20974836	*CDKAL1*	WcHtR	ADD	G	0.4095	-0.00696	0.04585
6	chr6:21006119	*CDKAL1*	WcHtR	ADD	G	0.4136	-0.00762	0.03029
6	chr6:21024305	*CDKAL1*	WcHtR	ADD	A	0.1215	-0.01457	0.005327
6	chr6:21063311	*CDKAL1*	WcHtR	ADD	G	0.2954	0.01022	0.00581
6	chr6:21102377	*CDKAL1*	WcHtR	ADD	G	0.2353	0.008117	0.0395
6	chr6:21115720	*CDKAL1*	WcHtR	ADD	A	0.4442	-0.00916	0.006996

6	chr6:21170579	*CDKAL1*	WcHtR	ADD	C	0.0914	-0.01373	0.02228
6	chr6:20824232	*CDKAL1*	Weight	ADD	A	0.3358	-1.31	0.0407
6	chr6:20916721	*CDKAL1*	Weight	ADD	A	0.4739	-1.255	0.03633
6	chr6:20932202	*CDKAL1*	Weight	ADD	G	0.1013	-2.118	0.03311
6	rs9350294	*CDKAL1*	Weight	ADD	A	0.4269	1.674	0.005873
6	chr6:21024416	*CDKAL1*	Weight	ADD	G	0.1031	-2.17	0.03167
6	chr6:21052580	*CDKAL1*	Weight	ADD	T	0.4629	-1.281	0.03185
6	chr6:21063311	*CDKAL1*	Weight	ADD	G	0.2954	1.799	0.005599
6	chr6:21099063	*CDKAL1*	Weight	ADD	A	0.4655	-1.319	0.02684
6	chr6:21102377	*CDKAL1*	Weight	ADD	G	0.2353	1.445	0.03672
6	chr6:21116822	*CDKAL1*	Weight	ADD	G	0.1573	-2.57	0.001778
6	chr6:21116972	*CDKAL1*	Weight	ADD	A	0.4527	-1.391	0.02039
6	chr6:21170579	*CDKAL1*	Weight	ADD	C	0.0914	-3.419	0.001173
10	chr10:114740147	*TCF7L2*	TGL	ADD	A	0.14	-0.1055	0.02476
10	chr10:114767131	*TCF7L2*	TGL	ADD	A	0.46	0.06579	0.04553
10	chr10:114787883	*TCF7L2*	TGL	ADD	A	0.19	-0.0875	0.03665
10	chr10:114808762	*TCF7L2*	TGL	ADD	G	0.49	0.07172	0.02812
10	chr10:114767131	*TCF7L2*	FPG	ADD	A	0.46	-0.1967	0.02289
10	chr10:114808762	*TCF7L2*	FPG	ADD	G	0.49	-0.231	0.007091
3	chr3:12282143	*PPARG*	BMI	ADD	A	0.12	-0.63	0.04322
3	chr3:12348324	*PPARG*	BMI	ADD	C	0.07	-1.219	0.002441
3	chr3:12281778	*PPARG*	WC	ADD	A	0.05	-1.962	0.04408
3	chr3:12348324	*PPARG*	WC	ADD	C	0.07	-2.468	0.004337
11	chr11:17333074	*KCNJ11*	TGL	ADD	G	0.1	0.1567	0.004517
11	chr11:17377424	*KCNJ11*	TGL	ADD	C	0.18	-0.1008	0.0189
11	chr11:17136712	*KCNJ11*	FPG	ADD	A	0.09	-0.3421	0.02046
10	chr10:114767131	*TCF7L2*	FPG	REC	A	0.46	-0.3306	0.02656
10	chr10:114808762	*TCF7L2*	FPG	REC	G	0.49	-0.3046	0.03323
10	chr10:114767131	*TCF7L2*	TGL	REC	A	0.46	0.1141	0.04393
3	chr3:12309991	*PPARG*	BMI	REC	G	0.47	0.8944	0.00971

3	chr3:12281778	*PPARG*	FPG	REC	A	0.05	2.417	0.04596
3	chr3:12194277	*PPARG*	TC	REC	C	0.22	-0.2211	0.0369
3	chr3:12264142	*PPARG*	TC	REC	G	0.49	0.119	0.03315
3	rs4135275	*PPARG*	TC	REC	G	0.18	0.3485	0.005604
8	chr8:118282520	*slc30a8*	TGL	REC	A	0.1	0.6508	0.007286
8	chr8:118282520	*slc30a8*	BMI	REC	A	0.1	3.344	0.02764
11	chr11:17136712	*KCNJ11*	BMI	REC	A	0.09	-3.356	0.01057
11	hg18_11_17365645	*KCNJ11*	WC	REC	G	0.42	-1.929	0.01764
11	chr11:17191160	*KCNJ11*	WC	REC	G	0.42	-1.744	0.03193
